# New BB0108, BB0126, BB0298, BB0323, and BB0689 Chromosomally Encoded Recombinant Proteins of *Borrelia burgdorferi* sensu lato for Serodiagnosis of Lyme Disease

**DOI:** 10.3390/pathogens13090767

**Published:** 2024-09-05

**Authors:** Weronika Grąźlewska, Tomasz Chmielewski, Beata Fiecek, Lucyna Holec-Gąsior

**Affiliations:** 1 Department of Biotechnology and Microbiology, Faculty of Chemistry, Gdańsk University of Technology, 80-233 Gdańsk, Poland; weronikagrazlewska@gmail.com; 2 Department of Parasitology and Diseases Transmitted by Vectors, National Institute of Public Health NIH—National Research Institute, 00-791 Warsaw, Poland; tchmielewski@pzh.gov.pl (T.C.); bfiecek@pzh.gov.pl (B.F.)

**Keywords:** *Borrelia burgdorferi* sensu lato, ELISA, Lyme disease, recombinant proteins, serodiagnosis

## Abstract

Five chromosomally encoded proteins, BB0108, BB0126, BB0298, BB0323, and BB0689, from *Borrelia burgdorferi* sensu lato (s.l.), were obtained in three variants each, representing the most common genospecies found in Europe (*Borrelia afzelii*, *Borrelia burgdorferi* sensu stricto (s.s.), and *Borrelia garinii*). The reactivity of these recombinant proteins with the IgM and IgG antibodies present in human serum was assessed using Western blot (WB) and the ELISA. In IgG-WB, the proteins exhibited varying reactivity, peaking at approximately 40–50% for BB0108 and BB0689. However, none of these proteins were recognized by specific antibodies in the IgM-WB. The sensitivity of IgG-ELISA based on three variants of BB0108 and BB0323 ranged from 71% to 82% and from 62% to 72%, respectively. Conversely, the specificity of both tested proteins was consistently above 82%. Tests utilizing single variants of BB0323 did not yield any diagnostic value in detecting IgM antibodies. However, BB0108 demonstrated recognition by antibodies present in 52% to 63% of the tested sera. These antigens appear advantageous due to the consistent reactivity observed across their variants. This observation suggests that appropriate selection of antigens conserved within *B. burgdorferi* s.l. could offer a solution to the issue of variable sensitivity encountered in serodiagnostic tests across Europe.

## 1. Introduction

*Borrelia burgdorferi* sensu lato (s.l.) is a group of bacteria that are an etiological factor of Lyme disease, the most prevalent tick-borne illness in the northern hemisphere. This bacterial group exhibits notable diversity, comprising 23 distinct representatives, of which 7 have been conclusively identified as pathogenic to humans (*Borrelia afzelii*, *Borrelia burgdorferi* sensu stricto (s.s.)*, Borrelia garinii*, *Borrelia bavariensis*, *Borrelia spielmanii*, *Borrelia mayonii, Borrelia lusitaniae*) [1,2]. In Europe, *B. afzelii*, *B. burgdorferi* s.s., and *B. garinii* are the predominant genospecies responsible for human infections, although instances *of B. bavariensis* and *B. spielmanii* infections have also been recorded [3]. Conversely, in North America, *B. burgdorferi* s.s. stands as the primary causative agent of Lyme disease, with occasional reports of infections caused by *B. mayonii* [3,4].

Symptoms of Lyme disease, apart from erythema migrans, which occurs only in 60–90% of patients, are not specific [5,6]. Therefore, the diagnosis is based mainly on laboratory methods. The cultural examination of *B. burgdorferi* s.l. in Barbour–Stönner–Kelly–H (BSK-H) and Kelly–Pettenkofer (MKP), although it is the standard goal, can only be applied in some particular situations, such as Acrodermatitis chronica atrophicans (ACA), and is not commonly used as it requires very experienced staff [7]. PCR, while less effective, is recommended primarily in the early stages of infection or in individuals with compromised immune systems. Its sensitivity is highest when involved tissues, such as the skin, cerebrospinal fluid, and synovial fluid, are used as input material. Blood samples are not suitable clinical specimens for diagnosing Lyme disease, as except for *B. mayonii*, *B. burgdorferi* s.l. is present in low concentrations in the blood. Additionally, the detection of DNA does not confirm whether an active infection is occurring [6,8,9]. The primary diagnostic approach for Lyme disease is a two-tiered serology assay, recommended by the CDC in 1995. Initially, a highly sensitive enzyme-linked immunosorbent assay (ELISA) is conducted, followed by confirmatory Western blot (WB) testing if the ELISA result is positive or equivocal [8,10].

The genome of *B. burgdorferi* s.l. is unique among bacteria, consisting of a linear chromosome and at least 21 plasmids, both linear and circular in nature. It is worth noting that not every isolate harbors a complete set of plasmids; their presence ranges from 7 to 21, with only the cp26, cp32, and lp54 plasmids universally present across all members of *B. burgdorferi* s.l. Consequently, individual isolates can exhibit significant variations in the genetic information encoded by plasmids [11,12,13].

Moreover, the antigenic profile of *Borrelia* varies depending on the host. Given the disparate physiological environments within ticks and mammals, *B. burgdorferi* s.l. has evolved a repertoire of genes that are selectively transcribed in response to specific hosts. This process initiates in response to the changing conditions during the tick’s blood meal, signaled by a rise in temperature and a decrease in pH. These environmental cues trigger the migration of spirochetes to the tick’s salivary glands, prompting the production of new proteins crucial for successful colonization [14].

Genes located on plasmids mainly encode proteins crucial for mediating bacterial interaction with host tissues, thus determining the pathogenicity and virulence of *B. burgdorferi* s.l. Isolates of *B. burgdorferi* s.l. not only exhibit antigenic diversity due to carrying different plasmid sets, but also demonstrate high variation in the amino acid sequences of proteins associated with a low degree of conservation of plasmid sequences between genospecies [13,15]. Therefore, although plasmid-encoded antigens are highly immunogenic, their diagnostic utility is often limited by their low reactivity with antibodies directed against different variants of the same antigens from other genospecies. This issue poses a particular challenge in Europe, where numerous genospecies of *B. burgdorferi* s.l. are pathogenic to humans [16,17,18].

A relatively well-conserved chromosome carries mainly the genes associated with fundamental metabolic processes. These include genes responsible for the synthesis of the cell wall, DNA, RNA and proteins, biosynthesis of membrane lipids and phospholipids, DNA repair, and nucleotide metabolism [15,19]. While these antigens exhibit lower immunogenicity when compared with plasmid-encoded proteins, they have not been subjected to extensive study by researchers. However, it is possible to identify among them those that induce the production of specific antibodies [16,20,21]. It is therefore possible that the use of these proteins in diagnostic tests may at least partly contribute to solving the problems related to the diversity of *B. burgdorferi* s.l. genospecies.

BB0108, BB0126, BB0298, BB0323, and BB0689 are chromosomally encoded proteins whose production increases during the establishment of mammalian infections. Additionally, BB0126, BB0323, and BB0689 are localized in the outer membrane of *B. burgdorferi* s.l., while BB0108 and BB0298 are located in the cell envelope. Therefore, there is a high probability that they play a role in pathogenesis and are presented to the immune system cells at the initial stage of infection, which may indicate their potential diagnostic utility [22].

In this study, for the first time, BB0108, BB0126, BB0298, BB0323, and BB0689 antigens from three genospecies of *B. burgdorferi* s.l. (*B. afzelii, B. burgdorferi* s.s., *B. garinii*) were biotechnologically produced with the use of the prokaryotic expression system of *Escherichia coli* and assessed for their reactivity with specific anti-*B. burgdorferi* s.l. antibodies.

## 2. Materials and Methods

### 2.1. Serum Samples

The study used 148 positive sera (100 for IgG and 48 for IgM) and 100 negative sera. Lyme disease serum samples were obtained from the National Institute of Public Health NIH (Warsaw, Poland). All were collected during a routine Lyme disease diagnosis. Anonymized information about each sample included only the date of collection and the titer of anti-*Borrelia* antibodies. The IgG and IgM levels were re-measured using *Borrelia* plus VlsE and *Borrelia* Select: recombinant antigens with OspC (Euroimmun, Lübeck, Germany), respectively. The presence of specific anti-*Borrelia* antibodies was further confirmed using a EUROLINE *Borrelia*-RN-AT (EUROLINE WB *Borrelia*, Euroimmun, Lübeck, Germany).

### 2.2. Construction of Recombinant Plasmids

Fragments of the BB0108, BB0126, BB0208, BB0323, and BB0689 genes were PCR-amplified from genome DNA of three *B. burgdorferi* s.l. genospecies (*B. burgdorferi* s.s. strain B31, *B. afzeli* strain PKo, *B. garinii* strain 20047 (DSMZ, Braunschweig, Germany)). When identical amino acid sequences were present at both ends of the amplified fragments, the same primers were used to amplify the gene from different genospecies (Supplementary Materials Table S1).

The PCR products were inserted into the BglII restriction site of pUET1 expression plasmid using In-Fusion^®^ HD Cloning Kit (Takara Bio USA, Inc., Mountain View, CA, USA) (Supplementary Materials Figures S1–S5) [23]. All the antigen nucleotide sequences were embedded in a frame between the His_6_-tag domains for the purification of the recombinant proteins using metal affinity chromatography. The nucleotide sequences of the recombinant plasmids were confirmed with DNA sequencing (Genomed, Poland).

### 2.3. Expression and Purification of Recombinant Proteins

All constructed recombinant plasmids were transformed into *Escherichia coli* BL21(DE3)pLysS. Cells transformed with recombinant plasmids were grown in LB broth supplemented with 100 μg/mL ampicillin and 34 μg/mL chloramphenicol to an optical density at λ = 600 nm of 0.4. Protein production was induced with isopropyl-β-D-thiogalactopyranoside (IPTG) at a final concentration of 1 mM, and cells were incubated with vigorous shaking at 37 °C for 18 h. Subsequently, cells were harvested via centrifugation. Proteins were purified in a one-step metal affinity chromatography in accordance with the manufacturer’s instructions (Novagen, Madison, WI, USA). The purification was carried out under native and denaturing conditions depending on whether the proteins were deposited in the dissolved cytoplasmic fraction or as inclusion bodies. Purity of the recombinant proteins was verified by 12% SDS-PAGE and quantified using Image Lab software 5.2.1 (Bio-Rad, Hercules, CA, USA). After purification, the His_6_-tags were not cleaved prior to serological analysis. Protein concentration was measured with a Bradford Assay Kit according to the manufacturer’s instructions (Bio-Rad, Hercules, CA, USA) using bovine serum albumin (BSA) as a standard.

### 2.4. Western Blot

Due to the similar masses of recombinant proteins, it was not feasible to test five antigens on a single test strip. Therefore, three different test strips were constructed, each containing protein preparations that distinctly varied in mass according to Table 1. SDS-PAGE was conducted on gels prepared using modified combs to have a single large well (Supplementary Materials Figure S21). Each enlarged well was loaded with 40 µg of recombinant protein. After electrophoretic separation, the proteins were transferred on a nitrocellulose membrane. The membrane was blocked with 5% non-fat skim milk in Tris-buffered saline with 0.1% Tween 20 (TBS-T) for 1 h at room temperature with constant shaking. The membrane was then washed three times with TBS-T and incubated with human serum samples, diluted at 1:200 for 1 h and 1.5 h for IgG and IgM detection, respectively. After the membrane was washed, it was treated for 1 h at room temperature with horseradish peroxidase-conjugated goat anti-human IgG and IgM antibodies (Jackson ImmunoResearch, Ely, UK), diluted at 1:75,000 and 1:50,000, respectively. After further washes, the reaction was developed by the addition of chemiluminescence peroxidase substrate (Immobilon Crescendo Western HRP substrate, Merck, Darmstadt, Germany), and the results were visualized using Image Lab software 5.2.1 (Bio-Rad, Hercules, CA, USA).

### 2.5. ELISA

MaxiSorp microtiter plates (Nunc, Waltham, MA, USA) were coated with individual variants of BB0108 and BB0323 antigens with a total of 1 μg per well in 50 mM carbonate buffer (pH 9.6). For the ELISA utilizing three antigen variants, microtiter plates were also coated with a total of 1 μg per well, with each antigen variant applied at an amount of 0.33 µg. After overnight incubation at 4 °C, the plates were washed four times with washing buffer (50 mM Tris; 0.88% NaCl; 0.1% Tween 20, pH 7.4). Plates were blocked for 1 h at 37 °C in blocking buffer (3% non-fat milk, 0.1 % Tween 20 in PBS). The plates were washed again and incubated for 1 h at 37 °C with the human serum diluted 1:100 in blocking buffer. After washing, peroxidase-labeled antibodies directed against human IgG and IgM (Jackson ImmunoResearch, Ely, UK) were added, diluted 1:32,000 and 1:16,000, respectively. O-phenylenediamine dihydrochloride (Substrate SIGMAFAST™ OPD, Sigma, Saint Louis, MO, USA) was used as a substrate for the detection of the formed immune complexes, and absorbances were measured at λ = 492 nm.

### 2.6. Statistical Analysis

For all data manipulation, analysis was conducted using GraphPad Prism software (Version 9, GraphPad Software Inc., San Diego, CA, USA). Fisher’s exact test was employed to assess the statistical significance of recombinant protein reactivity with antibodies present in two groups of sera in Western blots. The distinction in absorbance between negative and positive sera was evaluated using Student’s t-test. Statistical significance was established at a *p*-value below 0.05 for both tests. Receiver operating characteristic (ROC) analysis was performed to determine the area under the curve (AUC), optimal cut-off, sensitivity, and specificity of assays based on recombinant proteins. The optimal cut-off was identified as the absorbance value corresponding to the point on the ROC curve nearest to the (0, 1) corner [24].

## 3. Results

### 3.1. Construction of Recombinant Plasmids

In order to produce *B. burgdorferi* s.l. proteins, 15 recombinant plasmids carrying gene encoding selected antigens were successfully constructed. The characteristics of obtained plasmids and the proteins they encode are presented in Table 2. The full sequences of recombinant proteins are presented in the Supplementary Materials Figures S6–S20.

### 3.2. Expression and Purification of Recombinant Proteins

All the variants, BB0108, BB0126, BB0298, BB0323, and BB0689 proteins, were produced in *E. coli* BL21(DE3)pLysS. In this way, recombinant proteins with a molecular weight in the 23–50 kDa range were obtained (Table 2) (Supplementary Materials Figure S22). All proteins contained His_6_-tag domains at the N- and C-terminus which allowed for the purification of proteins using metal affinity chromatography. BB0108, BB0126, and BB0298 were produced in soluble form and purified under native conditions, while BB0323 and BB0689 were produced as inclusion bodies, thus requiring buffers containing 5 M urea for their purification. Proteins preparations were obtained with an electrophoretic purity greater than 98% (Supplementary Materials Figure S23). These prokaryotic expression systems produced 39–76 mg proteins per liter of culture (Table 2).

### 3.3. Western Blot

**Figure 1:**
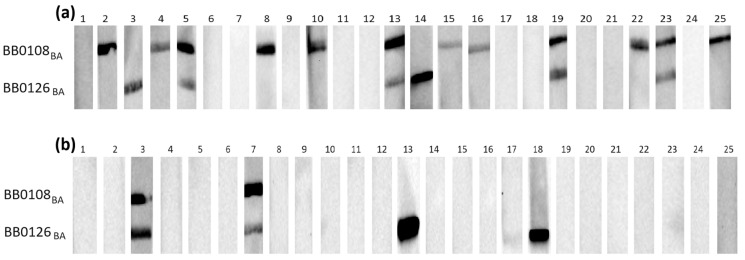
IgG Western blot results for BB0108_BA_ and BB0126_BA_: (**a**) incubation with 25 IgG-positive sera; (**b**) incubation with 25 negative sera.

**Figure 2:**
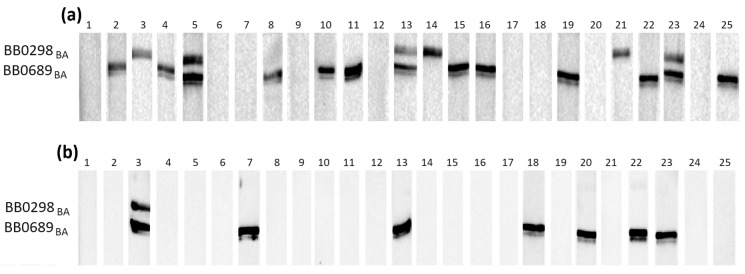
IgG Western blot results for BB0298_BA_ and BB0689_BA_: (**a**) incubation with 25 IgG-positive sera; (**b**) incubation with 25 negative sera.

**Figure 3:**
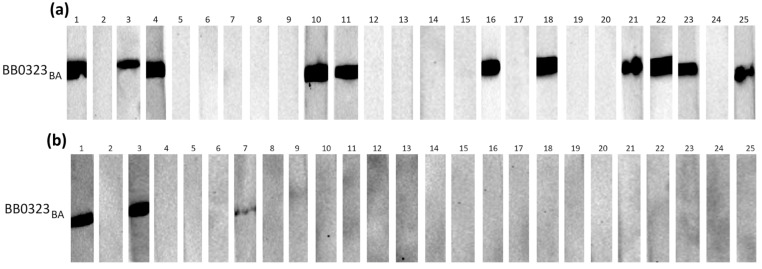
IgG Western blot results for BB0323_BA_: (**a**) incubation with 25 IgG-positive sera; (**b**) incubation with 25 negative sera.

The initial assessment of the reactivity of recombinant proteins with specific anti-*B. burgdorferi* IgG and IgM was conducted using three groups of serum samples, each comprising 25 individual samples. These groups included negative serum samples (IgG−, IgM−), IgG-positive samples (IgG+, IgM−), and IgM-positive samples (IgG−, IgM+). Figure 1, Figure 2 and Figure 3 depict representative results from Western blot analysis determining the reactivity of the tested antigens obtained from *B. afzelii* with class G antibodies (negative and IgG-positive sera).

Individual proteins exhibited varying reactivity with antibodies from human sera, with slight differences observed among protein variants originating from distinct genospecies. 

It appears that in most cases, the *B. burgdorferi* s.s.-derived protein was recognized by specific IgG less frequently than their *B. afzelii* and *B. garinii* equivalents (Table 3).

The most frequently recognized recombinant proteins by IgG in the positive sera were BB0108 and BB0689, with minimum reactivity levels of 44%, reaching a maximum of 52% for BB0689_BA_. All variants of BB0126 and BB0298 were very weakly reactive, recognized only by antibodies contained in approximately 20% of the tested sera. The specificity of antigen–antibody interactions was relatively high, at least 88%, except for BB0689, where the specificity dropped to 72%. After analysis using Fisher’s exact test, it was shown that a statistically significant difference in the reactivity of recombinant proteins with IgG contained in positive and negative sera occurs only for BB0108 (all variants), BB0323 (all variants), and BB0298_BG._

All tested proteins were poorly recognized by the IgM contained in serum samples (Table 4). The highest reactivity achieved for BB0108 from *B. afzelii* and *B. garinii* was 24%. The specificity of the IgM-WB was over 90% in all cases. Fisher’s exact test showed no statistically significant differences in the reactivity of all tested antigens with the IgM contained in the negative and positive sera (Table 4).

The overall sensitivity and specificity of the Western blot in detecting IgG and IgM when summing the results for the three variants of a given antigen remained unchanged relative to the most reactive one, except for IgG-WB-BB0689, where the sensitivity increased to 60% (15/25), while the specificity decreased to 64% (9/25).

Since solely WB based on BB0108 and BB0323 showed statistically significant differences in reactivity between the serum groups, only these proteins were selected for evaluating their diagnostic potential on a larger serum pool using the ELISA.

### 3.4. ELISA

**Figure 4:**
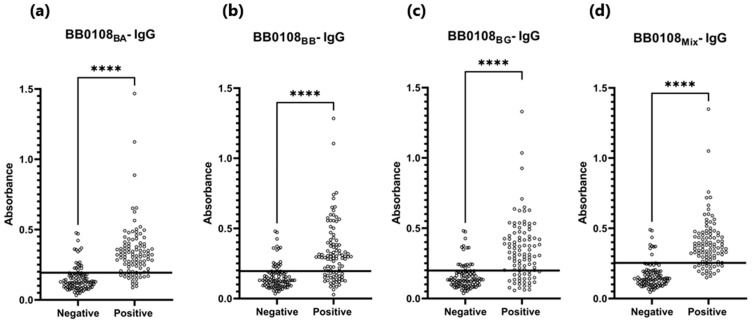
Absorbance for positive and negative sera: (**a**) IgG-ELISA-BB0108_BA_; (**b**) IgG- ELISA- BB0108_BB_; (**c**) IgG-ELISA- BB0108_BG_; (**d**) IgG-ELISA-BB0108_Mix_. The horizontal line marks the cut- off. ****—*p* ≤ 0.0001.

**Figure 5:**
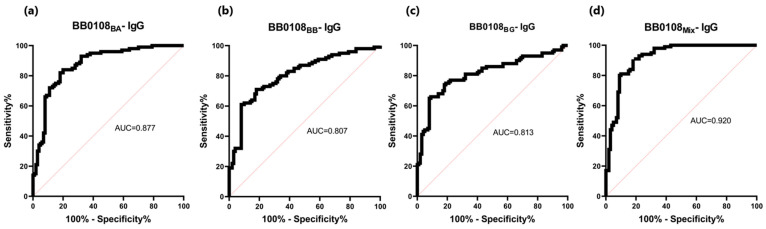
ROC analysis and AUC: (**a**) IgG-ELISA-BB0108_BA_; (**b**) IgG-ELISA-BB0108_BB_; (**c**) IgG- ELISA- BB0108_BG_; (**d**) IgG-ELISA-BB0108_Mix_.

**Figure 6:**
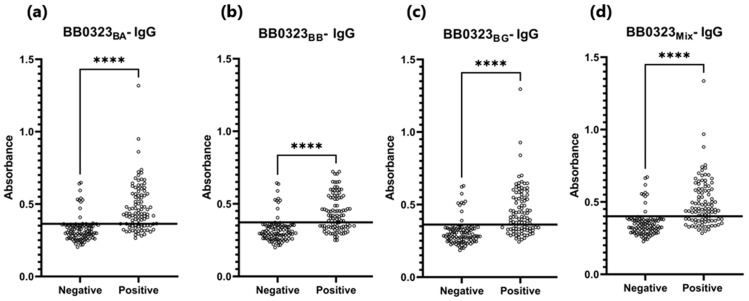
Absorbance for positive and negative sera: (**a**) IgG-ELISA-BB0323_BA_; (**b**) IgG- ELISA- BB0323_BB_; (**c**) IgG-ELISA- BB0323_BG_; (**d**) IgG-ELISA-BB0323_Mix_. The horizontal line marks the cut-off. ****—*p* ≤ 0.0001.

**Figure 7:**
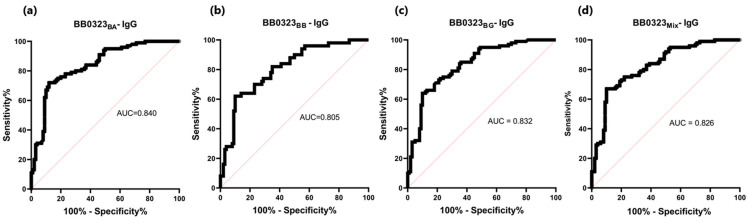
ROC analysis and AUC: (**a**) IgG-ELISA-BB0323_BA_; (**b**) IgG-ELISA-BB0323_BB_; (**c**) IgG- ELISA- BB0323_BG_; (**d**) IgG-ELISA-BB0323_Mix_.

**Figure 8:**
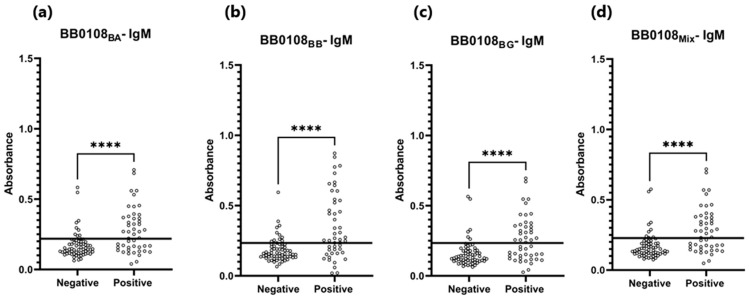
Absorbance for positive and negative sera: (**a**) IgM-ELISA-BB0108_BA_; (**b**) IgM-ELISA-BB0108_BB_; (**c**) IgM-ELISA-BB0108_BG_; (**d**) IgM-ELISA-BB0108_Mix_. The horizontal line marks the cut-off. ****—*p* ≤ 0.0001.

**Figure 9:**
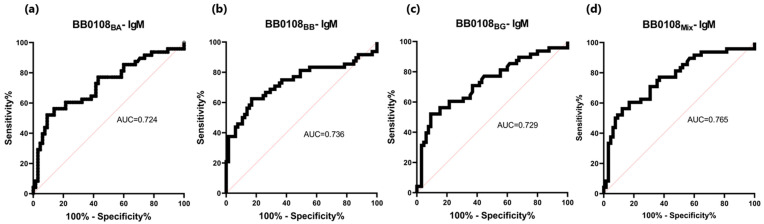
ROC analysis and AUC: (**a**) IgM-ELISA-BB0108_BA_; (**b**) IgM-ELISA-BB0108_BB_; (**c**) IgM-ELISA-BB0108_BG_; (**d**) IgM-ELISA-BB0108_Mix_.

**Figure 10:**
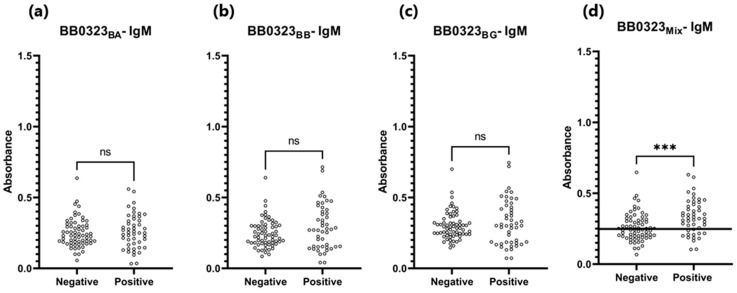
Absorbance for positive and negative sera: (**a**) IgM-ELISA-BB0323_BA_; (**b**) IgM-ELISA-BB0323_BB_; (**c**) IgM-ELISA-BB0323_BG_; (**d**) IgM-ELISA-BB0323_Mix_. The horizontal line marks the cut-off. ***—*p* ≤ 0.001. ns—*p* > 0.05.

**Figure 11:**
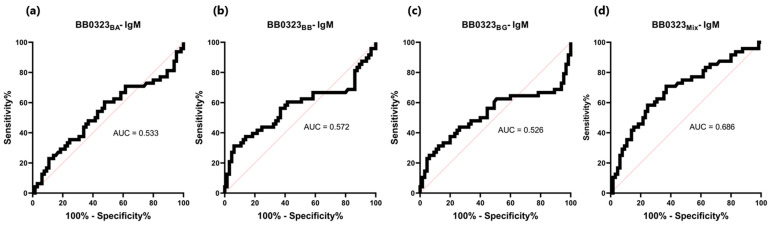
ROC analysis and AUC: (**a**) IgM-ELISA-BB0323_BA_; (**b**) IgM-ELISA-BB0323_BB_; (**c**) IgM-ELISA-BB0323_BG_; (**d**) IgM-ELISA-BB0323_Mix._

The BB0108 and BB0323 antigens showed differentiated reactivity with antibodies contained in human sera depending on the antigen variant (Table 5, Figure 4, Figure 5, Figure 6, Figure 7, Figure 8, Figure 9, Figure 10 and Figure 11). The sensitivity of the IgG-ELISA based on different variants of BB0108 ranged from 74% to 82%, with the highest value obtained for BB0108_BA_ (Table 5). The specificity of all IgG-ELISA-BB0108 was 82%. The area under curve (AUC) value ranged from 0.807–0.877 and again the highest value was achieved with BB0108_BA_ (Figure 5, Table 5). The use of a mixture of three variants of BB0108 recombinant proteins in the IgG-ELISA significantly increased the specificity of the assay and led to an increase in AUC (Figure 4 and Figure 5, Table 5). However, this did not affect the sensitivity of the assay.

**Table 5:** IgG-ELISA results.

Recombinant Protein	Optimal Cut-Off	Sensitivity [%]	Specificity [%]	AUC	Mean Absorbance	Median Absorbance
BB0108_BA_	0.193	82% (82/100) *	82% (18/100) *	0.877	P ^1^: 0.340 N ^2^: 0.153	P ^1^: 0.317 N ^2^: 0.128
BB0108_BB_	0.196	71% (71/100) *	82% (18/100) *	0.807	P ^1^: 0.323 N ^2^: 0.155	P ^1^: 0.297 N ^2^: 0.130
BB0108_BG_	0.199	74% (74/100) *	82% (18/100) *	0.813	P ^1^: 0.355 N ^2^: 0.157	P ^1^: 0.314 N ^2^: 0.132
BB0108_Mix_	0.254	80% (80/100) *	91% (9/100) *	0.920	P ^1^: 0.391 N ^2^: 0.166	P ^1^: 0.355 N ^2^: 0.141
BB0323_BA_	0.363	72% (72/100) *	88% (12/100) *	0.840	P ^1^: 0.473 N ^2^: 0.326	P ^1^: 0.433 N ^2^: 0.309
BB0323_BB_	0.372	62% (62/100) *	90% (10/100) *	0.805	P ^1^: 0.436 N ^2^: 0.321	P ^1^: 0.416 N ^2^: 0.299
BB0323_BG_	0.362	64% (64/100) *	90% (10/100) *	0.832	P ^1^: 0.450 N ^2^: 0.307	P ^1^: 0.413 N ^2^: 0.285
BB0323_Mix_	0.401	67% (67/100) *	90% (10/100) *	0.826	P ^1^: 0.492 N ^2^: 0.350	P ^1^: 0.452 N ^2^: 0.331

*—number of sera reactive with antigen/number of tested sera; AUC—area under the curve; ^1^—positive sera; ^2^—negative sera; _Mix_—a mixture of three variants of recombinant protein.

The sensitivity of the IgG-ELISA-BB0323 was the highest for BB0323_BA_ and the lowest for BB0323_BB_, at 72% and 62%, respectively. The specificity for all variants of the antigen oscillated around 90%, while the AUC reached the range of 0.805–0.840 (Figure 6 and Figure 7, Table 5).

The sensitivity of IgM detection was lower. It ranged for BB0108 variants from 52% to 63% (Table 5). However, unlike the IgG-ELISA here, BB0108_BB_ provided the highest sensitivity. AUC also decreased to 0.724–0.736 (Table 6, Figure 8 and Figure 9). However, a slightly higher specificity of these tests was observed, reaching 91% for BB0108_BG_. In the IgM-ELISA, using a mix of three protein variants did not significantly affect the sensitivity and specificity; instead, it led to an increase in AUC (Figure 9).

**Table 6:** IgM-ELISA results.

Recombinant Protein	Optimal Cut-Off	Sensitivity [%]	Specificity [%]	AUC	Mean Absorbance	Median Absorbance
BB0108_BA_	0.219	56% (27/48) *	86% (9/65) *	0.724	P ^1^: 0.277 N ^2^: 0.171	P ^1^: 0.259 N ^2^: 0.145
BB0108_BB_	0.234	63% (30/48) *	83% (12/65) *	0.736	P ^1^: 0.259 N ^2^: 0.183	P ^1^: 0.263 N ^2^: 0.159
BB0108_BG_	0.234	52% (25/48) *	91% (6/65) *	0.729	P ^1^: 0.264 N ^2^: 0.157	P ^1^: 0.269 N ^2^: 0.144
BB0108_Mix_	0.228	56% (27/48) *	86% (9/65) *	0.765	P ^1^: 0.287 N ^2^: 0.168	P ^1^: 0.246 N ^2^: 0.133
BB0323_BA_	No statistical difference in absorbance for positive and negative sera (*p* = 0.575)			0.533	P ^1^: 0.262 N ^2^: 0.250	P ^1^: 0.252 N ^2^: 0.232
BB0323_BB_	No statistical difference in absorbance for positive and negative sera (*p* = 0.052)			0.572	P ^1^: 0.294 N ^2^: 0.246	P ^1^: 0.273 N ^2^: 0.233
BB0323_BG_	No statistical difference in absorbance for positive and negative sera (*p* = 0.319)			0.526	P ^1^: 0.325 N ^2^: 0.301	P ^1^: 0.304 N ^2^: 0.284
BB0323_Mix_	0.248	60% (29/48) *	71% (29/65) *	0.686	P ^1^: 0.333 N ^2^: 0.261	P ^1^: 0.323 N ^2^: 0.243

*—number of sera reactive with antigen/number of tested sera; AUC—area under the curve; ^1^—positive sera; ^2^—negative sera; _Mix_—a mixture of three variants of recombinant protein.

Student’s *t*-test revealed non-statistically significant differences in absorbance between positive and negative sera in the IgM-ELISA based on single BB0323 variants (*p* > 0.05) (Table 5, Figure 10), with the acquired AUC values falling within the range of 0.526- 572 (Figure 11). When employing a protein mix in the IgM-ELISA, a statistically significant difference was observed in the absorbance levels between the two groups of sera.

## 4. Discussion

Despite its widespread use, serodiagnosis faces many challenges. The primary challenges stem from the complex antigenic structure of spirochetes. The high diversity of genospecies within *B. burgdorferi* s.l. and the low conservation level of amino acid sequences in their proteins mean that utilizing cell lysates from one genospecies in diagnostic tests may not be sufficient for accurate Lyme disease diagnosis [6,18,25]. Moreover, *B. burgdorferi* s.l. contains many proteins that are homologous to antigens present in other pathogens, which can lead to cross-reactivity. It is precisely these frequent nonspecific interactions that led to the introduction of a two-step serodiagnostic test [26,27,28].

A potential solution to these challenges could involve employing carefully selected recombinant proteins as antigens in serodiagnostic tests. Currently, Western blot tests frequently utilize biotechnologically produced antigens, facilitating improved standardization of antigen preparations and cost reduction. In Europe, to ensure better sensitivity of the ELISA, highly immunogenic *B. burgdorferi* s.l. recombinant antigens obtained from several genospecies are added to the whole cell lysate [25,29]. Because of its complex structure, the proteome of *B. burgdorferi* s.l. remains poorly understood. In fact, only a few of its antigens have been tested for diagnostic utility, with the majority being lowly conserved plasmid-encoded antigens, including DbpA, VlsE, BBK32, FlaB, OspA, OspC, OspB, OspF, and OspE. [30,31,32,33,34,35]. Studying the proteome of *B. burgdorferi* s.l. more closely may lead to the identification of highly conserved chromosome-encoded antigens with diagnostic utility, potentially eliminating the dependency of assay sensitivity on the infecting genospecies. The search for specific proteins can be carried out in different tissues or liquids (for example, in urine) with a high-definition tandem mass spectrometer [36].

For these reasons, in this study, it was decided to evaluate the diagnostic potential of five previously untested *B. burgdorferi* s.l. antigens whose genes are located on chromosomes and characterized by high-sequence conservation [22]. They were selected based on the literature data indicating their diagnostic potential. BB0323 is an antigen necessary for the transmission of spirochetes throughout the enzootic infection cycle. Additionally, BB0323 has been shown to trigger an immune response in both humans and mice [37].

BB0108 is a membrane lipoprotein, and two independent studies have confirmed the presence of specific antibodies in the sera of individuals with Lyme disease [21,38]. BB0689, localized on surface membrane, has been confirmed that it is an immunogen, as baboons infected with *B. burgdorferi* s.s. produced BB0689-specific bactericidal antibodies [39,40]. BB0126 and BB0298 are surface proteins, so theoretically they should be well-exposed to the host immune system [40]. Moreover, their up-regulation during tick blood feeding implies their potential involvement in the virulence and pathogenesis of *B. burgdorferi* s.l. [14]. It was decided to evaluate the reactivity of three variants of each of the antigens derived from *B. afzelii*, *B. burgdorferi* s.s., and *B. garinii* as these genospecies are the most common in Europe [41,42,43].

In this study, we utilized the efficient *E. coli* expression system developed in our laboratory, along with a simple purification method previously described [44,45], to obtain new recombinant proteins (BB0108, BB0126, BB0298, BB0323, BB0689) from three genospecies of *B. burgdorferi* s.l. These proteins were then employed in the IgG/IgM Western blot and IgG/IgM ELISA to assess their utility for a serological diagnosis of *Borrelia* infection in human sera. 

The initial assessment of reactivity for the new antigens, conducted using the WB test, revealed that the newly obtained proteins moderately react with antibodies present in human sera. The WB sensitivity reached its peak values for IgG-WB-BB0689; however, this antigen was also frequently recognized by antibodies in the negative sera (specificity range 72–76%). Therefore, it is likely that the relatively high sensitivity of the WB tests is primarily caused by cross-reactivity rather than specific interactions with anti-*B. burgdorferi* s.l. antibodies.

The most promising results at this stage of research were obtained for BB0108 and BB0323. Comparing these results to those obtained for recombinant proteins commonly used in commercial WB, it turned out that the reactivity of BB0108 and BB0323 was at a similar level as that obtained for IgG-DbpA_BA_ (45%; 9/20) in the study by Heikkilä et al. (2002) and exceeded that of the least reactive variant of this protein, *B. burgdorferi* s.s., which was 20% (4/20). However, when the results for the three DbpA variants in the study were added together, the overall sensitivity for detecting late-stage Lyme disease was 80% (16/20), significantly exceeding the value achieved in the cases of BB0108 and BB0323. In addition, WB based on DbpA was characterized by 100% specificity (0/20), while for BB0108 and BB0323, it was a maximum of 96% (1/25) and 92% (2/25), respectively [17]. WB based on the most reactive FlaA variant derived from *B. garinii* achieved a sensitivity of 75% (21/28) and a specificity of over 95% (1/23). When the reactivity of the three FlaA variants was added together, the sensitivity increased to 79% (22/28), and specificity decreased to 87% (3/23). FlaA from *B. burgdorferi* s.s. showed the lowest diagnostic utility, and the sensitivity decreased to 39% (11/28) with a specificity of 91% (2/23), which was a result comparable to that achieved for IgG-WB based on BB0108 and BB0323 [46].

As only BB0108 and BB0323 exhibited statistically significant differences in reactivity between negative and positive sera in the initial WB, it was decided to test these proteins in the ELISA assay on a large pool of sera. In the IgG-ELISA, the BB0108s were highly reactive with the antibodies contained in the positive sera. The sensitivity of the IgG-ELISA even reached above 80% for the most reactive variant of the antigen, which is similar to that obtained by DbpA in the late stages of Lyme disease [17,30]. However, it should be noted that the assay’s high sensitivity was not matched by its satisfactory specificity, as it was 82%. The use of the mixture of three variants of BB0108 in the ELISA did not improve the sensitivity of the test. It turned out to even be slightly lower than that obtained for the most reactive variant of the protein from *B. afzelii*. This can be explained by the lower content of BB0108_BA_ in the wells, as only 0.33 µg of BB0108_BA_ was used to coat the microtiter plates in this case, while it was 1 µg in the single protein assay. However, using the mixture positively affected the specificity and increased the AUC, significantly improving the diagnostic value of such an immunoenzymatic assay. This approach increased the overall difference in absorbance values between the two groups of sera. It was demonstrated that there is a more significant difference in mean absorbance and median between negative and positive sera in the ELISA assays using a mixture of variant proteins compared to those using single antigens. It is possible that by using three antigen variants, more anti-*B. burgdorferi* s.l. antibodies from positive sera recognized specific epitopes. This allowed the determination of a higher value of the optimal cut-off (0.254 compared to values oscillating around 0.195), leading to an increase in the specificity of the test, while only slightly lowering the sensitivity.

The sensitivity of the IgG-ELISA based on single variants of BB0323 was in the range of 62–72%. Similar to BB0108, the protein BB0323 obtained from *B. afzelii* was the most reactive, while the IgG-ELISA-BB0323_BB_ was characterized by the lowest sensitivity. Using the BB0323_Mix_ in the ELISA did not affect the diagnostic usefulness of the enzyme immunoassay. Although sensitivity compared to the ELISA based on single antigens obtained from *B. burgdorferi* s.s. and *B. garinii* increased, it did not reach as high a value as it did for BB0323_BA_. It is assumed that, similarly to the case of the BB0108s, it is related to the lower content of the most reactive protein variant (BB0323_BA_) in the microtiter plate wells. The specificity remained unchanged at 90%.

There was no statistically significant difference in absorbance between the positive and negative serums in the IgM-ELISA tests based on single variants of BB0323, and the obtained AUC values did not exceed 0.6 (0.533, 0.572, 0.526), indicating the lack of reactivity of this protein with this class of antibodies. Thus, tests based on single variants of BB0323 did not allow for distinguishing between IgM-negative and IgM-positive sera. The use of a mixture of three variants of BB0323 had an observable positive effect. The IgM-ELISA-BB0323_Mix_ showed a statistically significant difference in the absorbance of the positive and negative sera and the AUC increased to 0.686. Perhaps by utilizing a mixture of proteins from three genospecies in the ELISA, it was possible to detect infections with various representatives of *B. burgdorferi* s.l. in a single assay.

For the IgM-ELISA based on three variants of BB0108, statistically significant differences in absorbance were obtained for both sera groups, which suggests that BB0108 is recognized by specific IgM, which was not observed in the Western blot. This implies that conformational epitopes may play a major role in these interactions, as in the IgM-WB-BB0108 there was no statistically significant difference in the reactivity between negative and positive sera.

In both enzyme immunoassay tests (WB, ELISA), the obtained proteins were not recognized by specific IgM. This is likely due to the nature of IgM, as they are the primary antibodies produced during an immune response and typically exist at lower concentrations in the blood compared to IgG at their peak. Additionally, IgM levels in the blood decrease rapidly over time, and their affinity for antigens is generally lower as they are produced during the early stages of the immune response when it is still developing. Consequently, IgM antibodies have a narrower spectrum of antigen recognition, primarily targeting proteins and their fragments exposed during the early stages of infection [47,48,49,50,51].

Similarly, as in the previous studies, the sensitivity of ELISA and WB based on new recombinant *B. burgdorferi* s.l. antigens differed depending on the antigen variant used. It is important to note that differences in ELISA sensitivity were up to 11% and 10% for BB0108 and BB0323, respectively, while in the case of WB, they were even lower. Thus, they were not as meaningful as those observed in studies conducted for DbpA and OspC, where differences in ELISA sensitivity between variants in a single study were 25% and 50%, respectively [17,52]. Moreover, in studies by Heikkilä et al. (2002) [17] and Schulte-Spechtel et al. (2006) [53], it was observed that most of the sera were reactive with only one variant of the DbpA. In the present study, in most cases antibodies in the positive sera recognized all three antigen variants. The sensitivity of the ELISA and WB, taking into account all individual antigen variants, in most cases did not differ from that obtained for the most reactive form of the protein. Only in the case of IgG-BB0689s, when its three variants were considered, did the total WB sensitivity increase to 60% (15/25). The results suggest that in most cases, when antibodies against one variant of the antigen were present in the serum, they recognized its other forms. The observation that the most diverse reactivity of different protein variants concerns BB0689 is in line with the bioinformatic analysis, which showed that this antigen exhibited the lowest sequence conservation among those examined [22]. This indicates that the appropriate selection of conserved antigens may make their reactivity independent of the genospecies causing Lyme disease.

In most cases, similar to other research carried out on sera collected from different areas of Europe, the highest reactivity in the IgG class was shown by variants derived from *B. afzelii* or *B. garinii* [16,17,20,52]. This allows us to assume that infections with these genospecies were dominant among the patients from whom the sera were collected. However, these are only conjectures as no specific information was available. This observation is not entirely consistent with the research on the prevalence of *B. burgdorferi* s.l. genospecies in Poland, which state that *B. burgdorferi* s.s. is dominant [54,55]. However, it agrees with the reports that *B. afzelii* and *B. garinii* are the dominant genospecies in Europe [41,56]. It is noteworthy that this study, aimed at discerning the disparities in reactivity among distinct antigenic variants, has predominantly concentrated on three genospecies: *B. afzelii*, *B. burgdorferi* s.l., and *B. garinii*. Nevertheless, it is imperative to bear in mind the presence of *B. spielmanii* and *B. bavarsiensis* within Europe, potentially contributing to a broader spectrum of genospecies diversity [57].

It seems that the main parameter that reduces the diagnostic usefulness of BB0108 and BB0323 in the ELISA is their relatively low specificity compared to the previously characterized recombinant proteins. According to the literature, the specificity of the ELISA based on DbpA, VlsE, and OspC is very high and in many studies, it reached 100%, even when the immunoassays were performed on sera collected from individuals infected with pathogens that are the source of cross-reactions with *B. burgdorferi* s.l. [20,30,52,53,58,59,60,61]. This phenomenon may be due to the location of the genes encoding these proteins. DbpA, VlsE, and OspC are encoded by low-conserved plasmids and are mainly responsible for the pathogenesis and virulence of *B. burgdorferi* s.l. and are not directly necessary for the proper functioning of the bacterial cell. The *bb0108* and *bb0323* genes are located on the chromosome, where there are mainly genes that control the basic life functions of the cell, so there is a possibility that homologs of these proteins may be found in other organisms [15,19]. This means that the encoding of the protein by the chromosome ensures its high conservation and reactivity with antibodies directed against different genospecies of *B. burgdorferi* s.l.; however, on the other hand, it may be the cause of the increased cross-reactivity of these proteins [22].

It was noted that negative sera gave a false-positive overlap in this study, both for the antigens tested and antibody isotypes. The specificity of the IgG-ELISA-BB0108 for all antigen variants was 82%, in all cases; the same sera were responsible for the false-positive results. The same applies to the IgG-ELISA-BB0323. The same 10 sera were the source of cross-reactions in all BB0323 variants. In the IgM-ELISA-BB0108, the cross-reacting sera were also noted to have a partial overlap. However, it was not as clear as for the IgG-ELISA. It seems that such a correlation was not accidental. Unfortunately, the sera used in this study were not tested for antibodies against other pathogens. However, it seems worth checking whether these negative sera that gave false-positive results contain antibodies against pathogens that are the confirmed source of cross-reactions with *B. burgdorferi* s.l., i.e., *Treponema pallidum*, Relapsing fever *Borrelia*, Cytomegalo virus, Parvovirus B19, Epstein-Bar virus, *Anaplasma phagocytophilum*, and *Yersinia* [20,26,27,52,62,63,64,65,66]. It would be worthwhile to additionally examine sera for the presence of rheumatoid factors, the most common autoantibodies found in humans, as they may also recognize *B. burgdorferi* s.l. antigens [34].

In this study, the diagnostic utility of five newly chromosomally encoded recombinant proteins (BB0108, BB0126, BB0298, BB0323, BB0689) originating from three genospecies of *B. burgdorferi* s.l. (*B. afzelii*, *B. burgdorferi* s.s., *B. garinii*) was evaluated for the first time. Results obtained for two of these proteins, BB0108 and BB0323, suggest that conserved chromosomally encoded proteins might offer a promising alternative in the quest for new antigens for use in developing novel diagnostic tests. This is particularly significant in the context of creating immunoenzymatic tests that exhibit consistent utility regardless of the *Borrelia burgdorferi* genospecies causing the infection. Conducting fundamental research on chromosomally encoded proteins, followed by applied studies, constitutes a substantial contribution to a better understanding of the *B. burgdorferi* s.l. proteome, raising hopes for the development of new diagnostic tools. One avenue worth exploring is the utilization of selected fragments of these proteins in constructing chimeric proteins with diagnostic capabilities. For this purpose, experimental epitope mapping may hold significant value, enabling a precise understanding of antigen–antibody interactions and selection of the most applicable amino acids fragments [67]. By combining the robust immunogenicity of plasmid-encoded proteins with the conserved sequences of chromosomally encoded proteins, maybe there is potential to create immunoenzymatic tests of superior quality.

## 5. Conclusions

Highly conserved antigens encoded by the chromosome have been marginalized by scientists during the improvement of serodiagnosis of Lyme disease due to their lower immunogenicity when compared to antigens encoded by genes on plasmids. Despite their heightened immunogenicity, assays based on plasmid-encoded proteins have proven inadequate for diagnosing Lyme disease caused by various genospecies, as evidenced by numerous experiments. This limitation poses a significant challenge in Europe. This study demonstrates that conserved, chromosomally encoded antigens can also be recognized by anti-*B. burgdorferi* s.l. antibodies. Additionally, they exhibit reduced diversity in reactivity depending on the genospecies. Therefore, it seems justified to explore immunogenic proteins conserved within the *B. burgdorferi* s.l. group to develop universal tests that are easy and safe to produce and simpler to interpret. However, further effort is needed before ELISA assays, utilizing conserved chromosomally encoded recombinant proteins or chimeric/multivalent proteins as tools for epidemiological studies, become commercially available.

## Figures and Tables

**Table 1:** Composition of recombinant protein preparation used for the Western blot.

Test Strip No. 1	Test Strip No. 2	Test Strip No. 3
BB0108—44 kDa BB0126—30 kDa	BB0298s—32 kDa BB0698s—23 kDa	BB0323—50 kDa

**Table 2:** List of constructed plasmids and characterization of recombinant proteins.

Plasmid Name	Plasmid Length [bp]	Recombinant Protein	Protein Length [aa]	Native Protein aa Residues	Molecular Mass [kDa]	Production Efficiency [mg/L]
pUET1-BB0108_BA_	3839	BB0108_BA_	394	20–336	44.4	41
pUET1-BB0108_BB_	3839	BB0108_BB_	394	20–336	44.3	76
pUET1-BB0108_BG_	3839	BB0108_BG_	394	20–336	44.3	58
pUET1-BB0126_BA_	3408	BB0126_BA_	259	22–203	29.5	47
pUET1-BB0126_BB_	3408	BB0126_BB_	259	22–203	29.6	42
pUET1-BB0126_BG_	3408	BB0126_BG_	259	22–203	29.5	55
pUET1-BB0298_BA_	3489	BB0298_BA_	286	17–225	32.6	59
pUET1-BB0298_BB_	3489	BB0298_BB_	286	17–225	32.5	61
pUET1-BB0298_BG_	3489	BB0298_BG_	286	17–225	32.6	46
pUET1-BB0323_BA_	3933	BB0323_BA_	434	23–379	50.5	43
pUET1-BB0323_BB_	3927	BB0323_BB_	432	22–376	50	39
pUET1-BB0323_BG_	3924	BB0323_BG_	431	23–376	50.1	41
pUET1-BB0689_BA_	3237	BB0689_BA_	202	28–152	22.9	73
pUET1-BB0689_BB_	3237	BB0689_BB_	202	28–152	22.9	71
pUET1-BB0689_BG_	3237	BB0689_BG_	202	28–152	22.9	74

BA—*B. afzelii*; BB—*B. burgdorferi* s.s.; and BG—*B. garinii*.

**Table 3:** IgG-WB results.

Recombinant Protein	Sensitivity (*n* = 25)	Specificity (*n* = 25)	PPV ^1^	NPV ^2^	Statistically Significant (*p* < 0.05)
BB0108_BA_	48% (12/25) *	92% (2/25) *	86%	64%	Yes (*p* = 0.0036)
BB0108_BB_	40% (10/25) *	96% (1/25) *	91%	62%	Yes (*p* = 0.0046)
BB0108_BG_	44% (11/25) *	88% (3/25) *	79%	61%	Yes (*p* = 0.0255)
BB0108_BA+BB+BG_	48% (12/25) *	92% (2/25) *	86%	64%	Yes (*p* = 0.0036)
BB0126_BA_	24% (6/25) *	84% (4/25) *	60%	53%	No (*p* = 0.7252)
BB0126_BB_	20% (5/25) *	88% (3/25) *	63%	52%	No (*p* = 0.7019)
BB0126_BG_	16% (4/25) *	100% (0/25) *	100%	54%	No (*p* = 0.1099)
BB0126_BA+BB+BG_	24% (6/25) *	88% (3/25) *	67%	54%	No (*p* = 0.4635)
BB0298_BA_	24% (6/25) *	96% (1/25) *	86%	56%	No (*p* = 0.0983)
BB0298_BB_	20% (5/25) *	100% (0/25) *	100%	56%	No (*p* = 0.0502)
BB0298_BG_	24% (6/25) *	100% (0/25) *	100%	57%	Yes (*p* = 0.0223)
BB0298_BA+BB+BG_	24% (6/25) *	96% (1/25) *	86%	56%	No (*p* = 0.0983)
BB0323_BA_	44% (11/25) *	88% (3/25) *	79%	61%	Yes (*p* = 0.0255)
BB0323_BB_	36% (9/25) *	92% (2/25) *	82%	59%	Yes (*p* = 0.0374)
BB0323_BG_	44% (11/25) *	92% (2/25) *	85%	62%	Yes (*p* = 0.0083)
BB0323_BA+BB+BG_	44% (11/25) *	88% (3/25) *	79%	61%	Yes (*p* = 0.0255)
BB0689_BA_	52% (13/25) *	72% (7/25) *	65%	60%	No (*p* = 0.1482)
BB0689_BB_	44% (11/25) *	76% (6/25) *	65%	58%	No (*p* = 0.2321)
BB0689_BG_	48% (12/25) *	72% (7/25) *	63%	58%	No (*p* = 0.2436)
BB0689_BA+BB+BG_	60% (15/25) *	64% (9/25) *	63%	62%	No (*p*= 0.1564)

*—number of sera reactive with antigen/number of tested sera; ^1^—positive predictive value; ^2^—negative predictive value; _BA+BB+BG_—summed reactivity for three protein variants.

**Table 4:** IgM Western blot results.

Recombinant Protein	Sensitivity (*n* = 25)	Specificity (*n* = 25)	PPV ^1^	NPV ^2^	Statistically Significant (*p* < 0.05)
BB0108_BA_	24% (6/25) *	96% (1/25) *	86%	56%	No (*p* = 0.0983)
BB0108_BB_	20% (5/25) *	96% (1/25) *	83%	55%	No (*p* = 0.1895)
BB0108_BG_	24% (6/25) *	96% (1/25) *	86%	56%	No (*p* = 0.0983)
BB0108_BA+BB+BG_	24% (6/25) *	96% (1/25) *	86%	56%	No (*p* = 0.0983)
BB0126_BA_	16% (4/25) *	92% (2/25) *	67%	52%	No (*p* = 0.6671)
BB0126_BB_	8% (2/25) *	92% (2/25) *	50%	50%	No (*p* > 0.9999)
BB0126_BG_	8% (2/25) *	92% (2/25) *	50%	50%	No (*p* > 0.9999)
BB0126_BA+BB+BG_	16% (4/25) *	92% (2/25) *	67%	52%	No (*p* = 0.6671)
BB0298_BA_	8% (2/25) *	96% (1/25) *	67%	51%	No (*p* > 0.9999)
BB0298_BB_	12% (3/25) *	92% (2/25) *	60%	51%	No (*p* > 0.9999)
BB0298_BG_	8% (2/25) *	96% (1/25) *	67%	51%	No (*p* > 0.9999)
BB0298_BA+BB+BG_	12% (3/25) *	92% (2/25) *	60%	51%	No (*p* > 0.9999)
BB0323_BA_	8% (2/25) *	96% (1/25) *	67%	51%	No (*p* > 0.9999)
BB0323_BB_	4% (1/25) *	96% (1/25) *	50%	50%	No (*p* > 0.9999)
BB0323_BG_	8% (2/25) *	92% (2/25) *	50%	50%	No (*p* > 0.9999)
BB0323_BA+BB+BG_	8% (2/25) *	92% (2/25) *	50%	50%	No (*p* > 0.9999)
BB0689_BA_	20% (5/25) *	96% (1/25) *	83%	55%	No (*p* = 0.1895)
BB0689_BB_	12% (3/25) *	96% (1/25) *	75%	52%	No (*p* = 0.6092)
BB0689_BG_	12% (3/25) *	92% (2/25) *	60%	51%	No (*p* > 0.9999)
BB0689_BA+BB+BG_	20% (5/25) *	92% (2/25) *	71%	53%	No (*p* = 0.4174)

*—number of sera reactive with antigen/number of tested sera; ^1^—positive predictive value; ^2^—negative predictive value; _BA+BB+BG_—summed reactivity for three protein variants.

## Data Availability

All relevant data that support the findings are included in this published article/Supplementary Materials. Further inquiries can be directed to the corresponding author.

## Supplementary Materials

The following supporting information can be downloaded at: https://www.mdpi.com/article/10.3390/pathogens13090767/s1, Table S1. Sequences of the primers used to amplify the *B. burgdorferi* s.l. genes (sequence complementary to the gene is underlined, bold nucleotides have been inserted to shift the ORF), Figure S1: Scheme of pUET1-BB0108_BA_ construction using the In-Fusion system, Figure S2: Scheme of pUET1-BB0126_BA_ construction using the In-Fusion system, Figure S3: Scheme of pUET1-BB0298_BA_ construction using the In-Fusion system, Figure S4: Scheme of pUET1-BB0323_BA_ construction using the In-Fusion system, Figure S5: Scheme of pUET1-BB0689BA construction using the In-Fusion system, Figure S6: Amino acid sequence of the BB0108_BA_ monovalent recombinant protein, Figure S7: Amino acid sequence of the BB0108_BB_ monovalent recombinant protein, Figure S8: Amino acid sequence of the BB0108_BG_ monovalent recombinant protein, Figure S9: Amino acid sequence of the BB0126_BA_ monovalent recombinant protein, Figure S10: Amino acid sequence of the BB0126_BB_ monovalent recombinant protein, Figure S11: Amino acid sequence of the BB0126_BG_ monovalent recombinant protein, Figure S12: Amino acid sequence of the BB0298_BA_ monovalent recombinant protein, Figure S13: Amino acid sequence of the BB0298_BB_ monovalent recombinant protein, Figure S14: Amino acid sequence of the BB0298_BG_ monovalent recombinant protein, Figure S15: Amino acid sequence of the BB0232_BA_ monovalent recombinant protein, Figure S16: Amino acid sequence of the BB0323_BB_ monovalent recombinant protein, Figure S17: Amino acid sequence of the BB0323_BG_ monovalent recombinant protein, Figure S18: Amino acid sequence of the BB0689_BA_ monovalent recombinant protein, Figure S19: Amino acid sequence of the BB0689_BB_ monovalent recombinant protein, Figure S20: Amino acid sequence of the BB0689_BG_ monovalent recombinant protein, Figure S21: SDS-PAGE of *B. burgdorferi* s.l. recombinant proteins in polyacrylamide gel prepared with the use of modified combs, Figure S22: Production of recombinant proteins. SDS-PAGE of proteins contained in *E. coli* BL21(DE3)pLysS whole cell lysates, Figure S23: Results of purification of recombinant proteins.
