# Supplementary material for: New BB0108, BB0126, BB0298, BB0323, and BB0689 Chromosomally Encoded Recombinant Proteins of Borrelia burgdorferi sensu lato for Serodiagnosis of Lyme Disease
Source: Pathogens. 2024 Sep 5;13(9):767. doi: 10.3390/pathogens13090767 (PMC11434722; doi:10.3390/pathogens13090767)
Supplement: Supplementary file 1 [file pathogens-13-00767-s001.zip › pathogens-3037893-supplementary.pdf]

Table S1. Sequences of the primers used to amplify the *B. burgdorferi* s.l. genes (sequence complementary to the gene is underlined, bold nucleotides have been inserted to shift the ORF)

Primer Name	Primer Sequence	Length (bp)	GC [%]	Tm [°C]
ForBB0108BG/BA/BB	TGGACAGCCCAGATC**CT**CAAAATACTCCTGTTGCTATTATTAATCTATATAAAAATGAAA	60	32	66
RevBBA0108BG/BB	ATCGGTACCCAGATCTTTTAGACTAGAATCCAAGATTTGTATATTTGCAGACTTG	55	36	66
RevBBA0108BA	ATCGGTACCCAGATCTTTTAGACTAGAATCCAAAATTTGTATACTTGCAGACTTG	55	36	67
ForBB0126BB/BG	TGGACAGCCCAGAT**CT**TGTTGTTTTTTATAATTCTTTAGGCAAGGATTATGTAAAGAGT	60	32	66
ForBB0126BA	TGGACAGCCCAGAT**CT**TGTTGTTTTTTATAATTCTTTAAGTAAAGATTATGTAAAGAGTGGTGG	64	43	69
RevBB0126BA/BB/BG	ATCGGTACCCAGATCATTTTGCTTAAGTTCTAAAATTTTATTATTTGCCATAT	55	36	66
ForBB0298BA	TGGACAGCCCAGATC**GT**GGTAGCGAATCTAAAGAAAAATTGAATCTTGG	49	43	69
ForBB0298BB	TGGACAGCCCAGATC**GT**GGCAATGAATCTAAAGAAAAATCAAATCTTGGT	50	40	69
ForBB0298BG	TGGACAGCCCAGATC**GT**GGTAGTGAATCTAAAGAAAAATTGAATCTTGGG	50	42	69
RevBB0298BA/BB/BG	ATCGGTACCCAGATCGATCCTAGAAACACCTTCTTTTTGCTCT	43	44	68
ForBB0323BA	TGGACAGCCCAGATC**TT**AAAACGCCTCCGG	30	63	66
ForBB0323BB	TGGACAGCCCAGATC**AT**ACGCCTCCAGAATCAAGAGAG	38	53	70
ForBB0323BG	TGGACAGCCCAGATC**AT**ACAACGCCTCCAGAAGCAAG	37	54	71
RevBB0323BA	ATCGGTACCCAGATCTTTAGCAGGAATTATTATTTTCCAGTTAGAAT	47	34	64
RevBB0323BB	ATCGGTACCCAGATCTTTGGCAGGAATTATTATCTTCCAGTTAGAATGAATTAGA	55	36	67
RevBB0323BG	ATCGGTACCCAGATCTTTAGCAGGAATTATTATCTTCCAGTTGGAATGAATCA	53	38	67
ForBB0689BA/BB/BG	TGGACAGCCCAGATC**GC**GAAGATATGAAAATTCTATATTCAGAAAT	46	37	66
RevBB0689BA/BB/BG	ATCGGTACCCAGATCTTTTCTTTTTCCAAAAAGAACTACAAATATATCTATATT	54	28	63

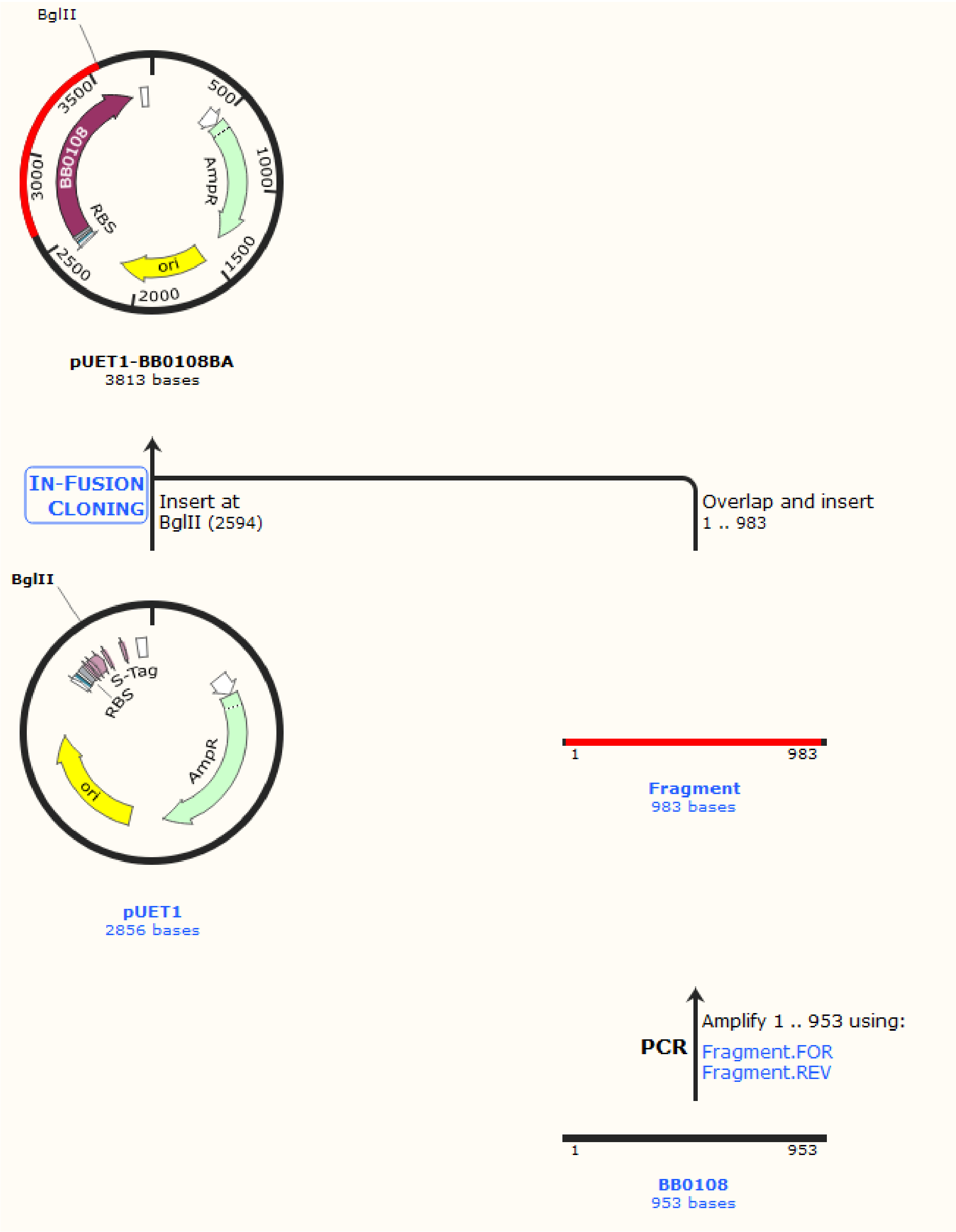

Figure S1. Scheme of pUET1-BB0108BA construction using the In-Fusion system.

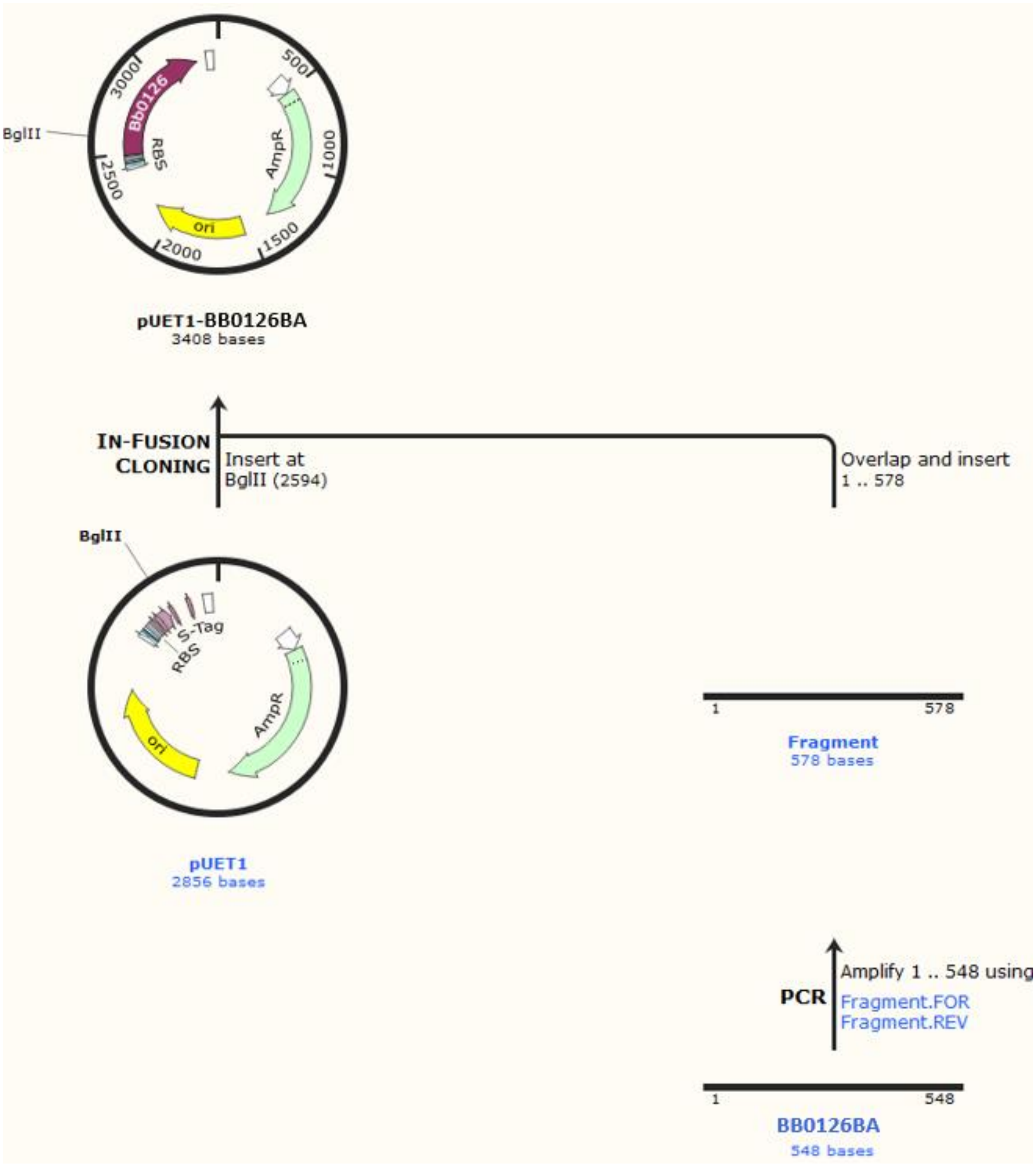

Figure S2. Scheme of pUET1-BB0126BA construction using the In-Fusion system.

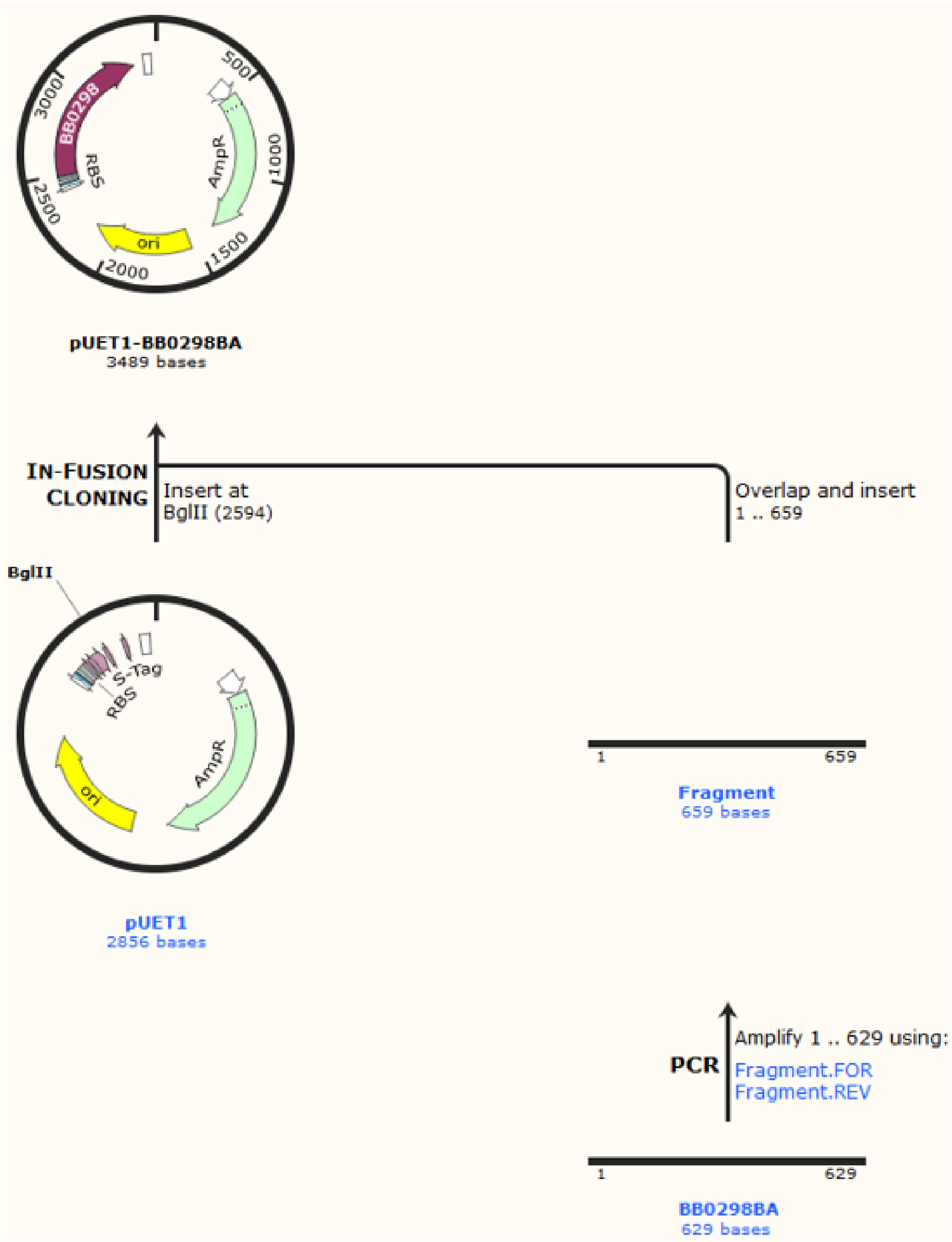

Figure S3. Scheme of pUET1-BB0298BA construction using the In-Fusion system.

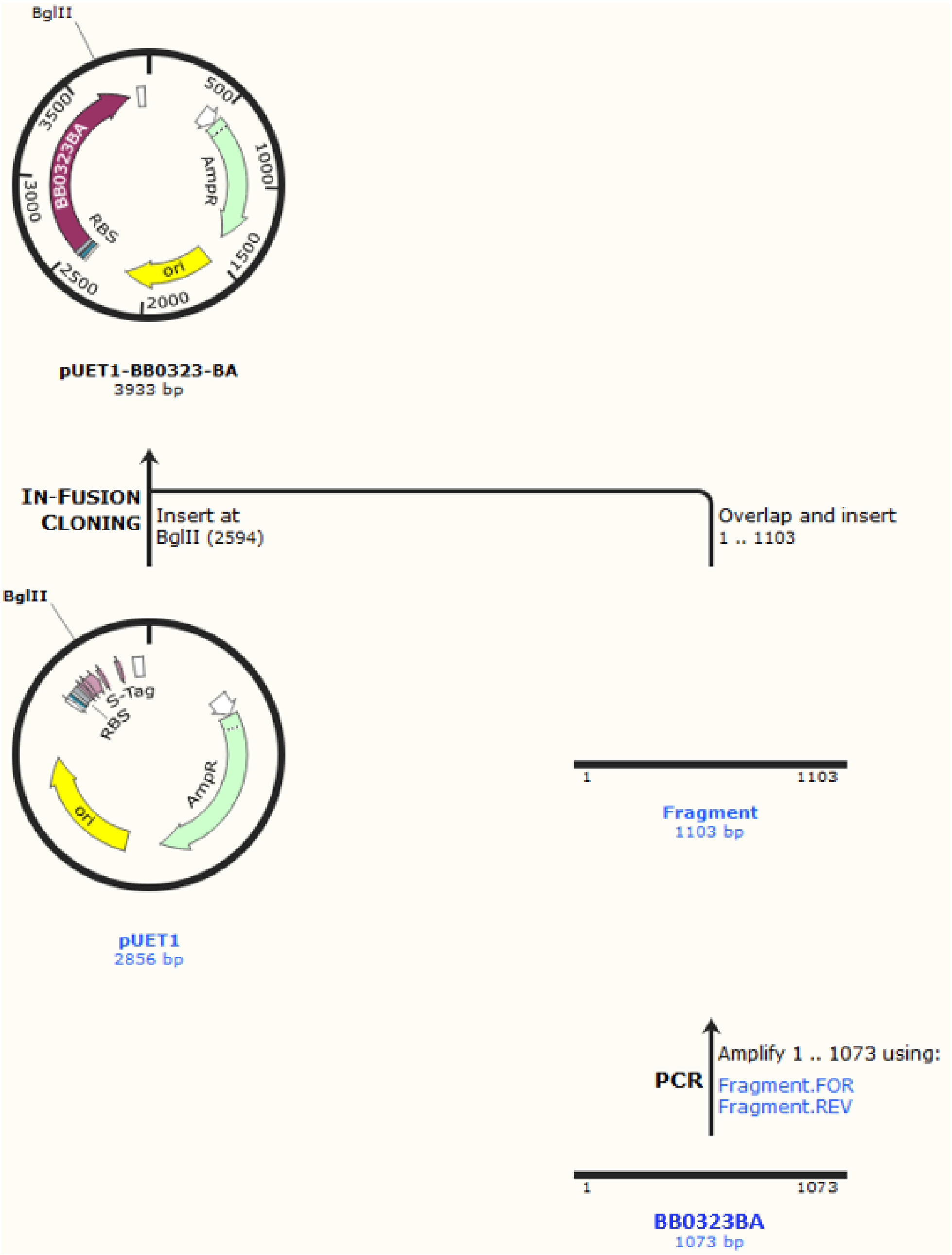

Figure S4. Scheme of pUET1-BB0323BA construction using the In-Fusion system.

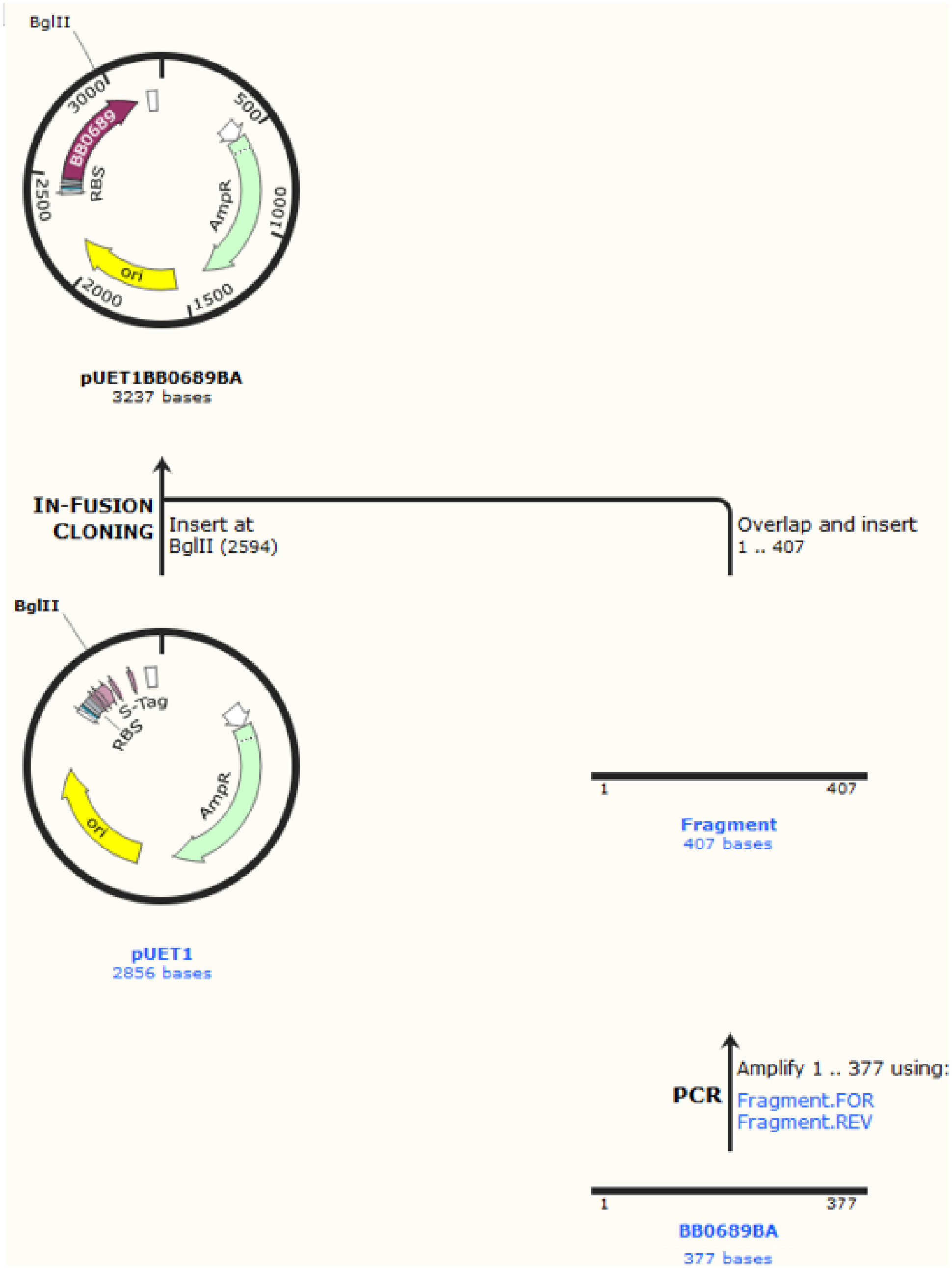

Figure S5. Scheme of pUET1-BB0689$_{BA}$ construction using the In-Fusion system.

1-60 MHHHHHHSSG LVPRGSGMKE TAAAKFERQH MDSPDPQNTP VAIINLYKNE IITKTSFDSK
61-120 VDIFKKTQGR DLTAAEKKQV LQVLIADVLF SQEASKQGIK ISDDEVMQTI RTQFGLVNFT
121-180 DEQIKQMIEK QGTNWGELLS SMKRSLSSQK LVLKQAQPRF SEVKTPSEKE IIEYYEANKT
181-240 RFVNPDISRV SHVFFSTKDK KRSDVLDQAK NILSQIRSKK ITFEEAVRKY SNDESSKAKN
241-300 GDLGFLSRDD QNAQNLLGPD FIKEVFNFNK GDISSPIASK EGFHIVKVTE KYAQRFLGLN
301-360 DKVSPATDLI VKDAIRNNMV NIQQQQIVVQ VQQDVYGKLN KSASIQILDS SLKDLGTDDD
361-394 DKSPGFSSTM AISDPNSSSV DKLAAALEHH HHHH

Figure S6. Amino acid sequence of the BB0108$_{BA}$ monovalent recombinant protein. The sequence derived from the pUET1 vector is marked in black and the His-tag sequence within it is marked in green; the S-Tag domain in orange; fragment of the BB0108$_{BA}$ is marked in blue.

1-60 MHHHHHHSSG LVPRGSGMKE TAAAKFERQH MDSPDPQNTP VAIINLYKNE IITKTSFDSK

61-120 VDIFKKTQGR DLTAAEKKQV LQVLIADVLF SQEASKQGIK ISDDEVMQTI RTQFGLVNFT

121-180 DEQIKQMIEK QGTNWGELLS SMKRSLSSQK LVLKQAQPRF SEVKTPSEKE IIEYYEANKT

181-240 RFVNPDISRV SHVFFSTKDK KRSDVLDQAK NILSQIRSKK ITFEEAVRKY SNDESSKAKN

241-300 GDLGFLSRDD QNAQNLLGPD FIKEVFNFNK GDISSPIASK EGFHIVKVTE KYAQRFLGLN

301-360 DKVSPATDLI VKDAIRNNMV NIQQQIVVQ VQQDVYGKLN KSASIQILDS SLKDLGTDDD

361-394 DKSPGFSSTM AISDPNSSSV DKLAAALEHH HHHH

Figure S7. Amino acid sequence of the BB0108$_{BB}$ monovalent recombinant protein. The sequence derived from the pUET1 vector is marked in black and the His-tag sequence within it is marked in green; the S-Tag domain in orange; fragment of the BB0108$_{BB}$ is marked in blue.

1-60 MHHHHHHSSG LVPRGSGMKE TAAAKFERQH MDSPDPQNTP VAIINLYKNE IITKTGFDSK

61-120 VDIFKKTQGR DLTAAEKKQV LQVLIADVLF SQEASKQGIK ISDDEVMQTI RTQFGLVNLT

121-180 DEQIKQMIEK QGTNWGELLA SMKRSLSSQK LVLKQAQPRF SEVKTPSEKE IVEYYEANKT

181-240 KFVNPDISRV SHVFFSTKDK KRSEVLDQAK NILSQIRSKK ITFEEAVRKY SNDESSKAKN

241-300 GDLGFLSRGD QNAQNLLGAD FIKEVFNFNK GDISSPIASK EGFHIIKVTE KYAQRFLGLN

301-360 DKVSPTTDLI VKDAIRNNMV NIQQQIVVE VQQDVYSKLN KSANIQILDS SLKDLGTDDD

361-394 DKSPGFSSTM AISDPNSSSV DKLAAALEHH HHHH

Figure S8. Amino acid sequence of the BB0108$_{BG}$ monovalent recombinant protein. The sequence derived from the pUET1 vector is marked in black and the His-tag sequence within it is marked in green; the S-Tag domain in orange; fragment of the BB0108$_{BG}$ is marked in blue.

1-60 MHHHHHHSSG LVPRGSGMKE TAAAKFERQH MDSPDLVVFY NSLSKDYVKS GGEIVENLEK

61-120 DLNDYLKEND AKEREKISLR IKELILKEKE ISSYFISRFY LAKAVYLQSQ SQYDEAIKDL

121-180 DIVIKAKGIE SEIAFINKAT IYEKMGLKED ALLVYEDLIK NTSLGFLKVR ALLSKAILIE

181-240 EKDKDLAVKV YEEIVKFPYE NNLYINIANN KILELKQNDL GTDDDDKSPG FSSTMAISDP

241-259 NSSSVDKLAA ALEHHHHHH

Figure S9. Amino acid sequence of the BB0126$_{BA}$ monovalent recombinant protein. The sequence derived from the pUET1 vector is marked in black and the His-tag sequence within it is marked in green; the S-Tag domain in orange; fragment of the BB0126$_{BA}$ is marked in blue.

1-60 MHHHHHHSSG LVPRGSGMKE TAAAKFERQH MDSPDLVVFY NSLGKDYVKS GGEIVENLEK

61-120 DLNDYLKEND AKEREKIFLR IRELISKEKE ISSYFISRFY LARAVYFQSQ AQYDEAIKDL

121-180 DIVIKAKGIE SEIAFLNKAA VYEKMGLKED ALLVYEDLIN STSLDFLKVR ALLSKAILIE

181-240 EKDKELAVKV YEEIVKFPYE NNLYINMANN KILELKQNDL GTDDDDKSPG FSSTMAISDP

241-259 NSSSVDKLAA ALEHHHHHH

Figure S10. Amino acid sequence of the BB0126$_{BB}$ monovalent recombinant protein. The sequence derived from the pUET1 vector is marked in black and the His-tag sequence within it is marked in green; the S-Tag domain in orange; fragment of the BB0126$_{BB}$ is marked in blue.

1-60 MHHHHHHSSG LVPRGSGMKE TAAAKFERQH MDSPDLVVFY NSLGKDYVKS GGEIVENLEK

61-120 DLNDYLKEND TKEREKISLR IKELISKEKE ISSYFISRFY LARAFYLQSQ AQYDEAIKDL

121-180 DIVIKAKGIE SEIAFINKAA VYEKMGLKED ALLVYEELIN STSLGFLKVR ALLSKAILIE

181-240 EKDKDLAVKV YEEIVKFPYE NNLYINIANN KILELKQNDL GTDDDDKSPG FSSTMAISDP

241-259 NSSSVDKLAA ALEHHHHHH

Figure S11. Amino acid sequence of the BB0126$_{BG}$ monovalent recombinant protein. The sequence derived from the pUET1 vector is marked in black and the His-tag sequence within it is marked in green; the S-Tag domain in orange; fragment of the BB0126$_{BG}$ is marked in blue.

```
1-60    MHHHHHHSSG LVPRGSGMKE TAAAKFERQH MDSPDRGSES KEKLNLGLRL RELEISGGGS
61-120  ESKIEVYKEF IEKEDKNILK IVNSIDKKAR FFNLIGLEFF KLSQYGPAIE YFTKNLEINS
121-180 DNYLSHFYVG VASYNLAKNL RVKDEVEKYI ILAENSFLKS LSIRDDFKES LFAISNMYVY
181-240 DLDKQLEAKN YLNKLEDMGE DYFEFFMLRG ANYYSLGDLG NAILFYDKAS KNASTEEQKE
241-286 GVSRIDLGTD DDDKSPGFSS TMAISDPNSS SVDKLAAALE HHHHHH
```

Figure S12. Amino acid sequence of the BB0298BA monovalent recombinant protein. The sequence derived from the pUET1 vector is marked in black and the His-tag sequence within it is marked in green; the S-Tag domain in orange; fragment of the BB0298BA is marked in blue.

```
1-60    MHHHHHHSSG LVPRGSGMKE TAAAKFERQH MDSPDRGNES KEKSNLGLRL RELEISGGGS
61-120  ESKIEVYKEF IEKEDKNILK IVNSIDKKAR FFNLIGLEFF KLGQYGPAIE YFAKNLEINP
121-180 NNYLSHFYIG VASYNLAKNL RVKDEVEKYI ILAENSFLKS LSIRDDFKDS LFAISNMYVY
181-240 DLDKQLEAKN YLNKLGDMGE DYFEFLMLRG ANYYSLGDLG NAILFYDKAS KKASTEEQKE
241-286 GVSRIDLGTD DDDKSPGFSS TMAISDPNSS SVDKLAAALE HHHHHH
```

Figure S13. Amino acid sequence of the BB0298BB monovalent recombinant protein. The sequence derived from the pUET1 vector is marked in black and the His-tag sequence within it is marked in green; the S-Tag domain in orange; fragment of the BB0298BB is marked in blue.

```
1-60    MHHHHHHSSG LVPRGSGMKE TAAAKFERQH MDSPDRGSES KEKLNLGLRL RELEISGGGS
61-120  ESKIEVYKEF IEKEDKNILK IVNSIDKKAR FFNLIGLEFF KLGQYGPAIE YFVKNLEINS
121-180 NNYLSHFYIG VASYNLAKNL RVKDEVEKYI ILAENSFLKS LSIRDDFKDS LFAISNMYVY
181-240 DLDKQLEAKN YLNKLDDMGE DYFEFFMLRG ANYYSLGDLG NAILFYEKAS KNASTEEQKE
241-286 GVSRIDLGTD DDDKSPGFSS TMAISDPNSS SVDKLAAALE HHHHHH
```

Figure S14. Amino acid sequence of the BB0298BG monovalent recombinant protein. The sequence derived from the pUET1 vector is marked in black and the His-tag sequence within it is marked in green; the S-Tag domain in orange; fragment of the BB0298BG is marked in blue.

```
1-60    MHHHHHHSSG LVPRGSGMKE TAAAKFERQH MDSPDRKTPP EARESKNAKI AQPNNEIFQL
61-120  RDLKDIKNEL IRERGHLFYS KEFNEAERLE EAMKQNFSKK KAKEGNEIAL KVLERYKTII
121-180 KETKEKKEKT NYLKENIEKY LNDAEANEAY IWIPLEIDEV NNLYFEATRK YKNYDLDNAL
181-240 DMYSKAFNRA QQAAKNAKEA KALKETDERM YKQLKALEAA SNLPVYSNNK LIKPSPWNGR
241-300 AFIKERNGHL NLLNINAEDT YFLGETKTST PIVLAYEEKM EIAKTSNPQE QFKTLELIEQ
301-360 SRKLWEKGVE AKNVKNFRLA NELFLESARY LEAYQSNASS ELYVIKIGNT LWGISKKLYN
361-420 DPYLWPKIWF ANRQKIQNPD LIHSNWKIII PAKDLGTDDD DKSPGFSSTM AISDPNSSSV
421-434 DKLAAALEHH HHHH
```

Figure S15. Amino acid sequence of the BB0323BA monovalent recombinant protein. The sequence derived from the pUET1 vector is marked in black and the His-tag sequence within it is marked in green; the S-Tag domain in orange; fragment of the BB0323BA is marked in blue.

```
1-60    MHHHHHHSSG LVPRGSGMKE TAAAKFERQH MDSPDQTPPE SRESKNAKIA QPDNKNFQLR
61-120  DIKDIKNELI RERGHLFYSK EFNEAERLEE AMKQSFSKKK AIEGNEIALK VLERYKTIIR
121-180 ETREKKEKTN YLKENIEKYL NDAEANEAYI WIPLEIDEVN NLYFEATRKY KNYDLDNALD
181-240 MYSKAFNRAQ QAAKNAKEAK ALKETDERMY KQLKALEAAS NLPIYSNNKL IKPSPWNGRA
241-300 FIKERNSHLN LLNTNKDTYL LGEAEISIPI VLAYEEKVEI AKNSKPQEQF KTLELIERSR
301-360 TLWEKGVEAK NVKNFRLANE LFLESARYLE AYQSNASSEL YVIKIGNTLW GISKKLYNDP
361-420 YLWPKIWFAN RQKIQNPDLI HSNWKIIIPA KDLGTDDDDK SPGFSSTMAI SDPNSSSVDK
421-432 LAAALEHHHH HH
```

Figure S16. Amino acid sequence of the BB0323BB monovalent recombinant protein. The sequence derived from the pUET1 vector is marked in black and the His-tag sequence within it is marked in green; the S-Tag domain in orange; fragment of the BB0323BB is marked in blue.

1-60 MHHHHHHSSG LVPRGSGMKE TAAAKFERQH MDSPDHTTPP EARENKNAKI AKLDTKSFEL
61-120 RDIKDIKNEL IKERGHLFYS KEFNEAEKLE EAMKQNFSKK KAKEINEIAL KVIERYKTII
121-180 KETREKKEKV NYLKENIEKY LNDAEANEAY IWIPLEIDEV NNLYFEATRK YKNYDLDDAL
181-240 GMYSKAFNRA QQAAKNAKEA KALKETDERM YKQLKALEAA SNLPIYSNNK LIKPSPWNGR
241-300 AFIKERNSRL NLLNINEDTY LLGETETPIV LAYTEKLEIA KNSKPQEQFK TLELIERSRK
301-360 LWEKGVEAKH VKNFRLANEL FLESARYLEA YQSNASSELY VIKIGNTLWG ISKKLYNDPY
361-420 LWPKIWFANR QKIQNPDLIH SNWKIIIPAK DLGTDDDDKS PGFSSTMAIS DPNSSSVDKL
421-431 AAALEHHHHH H

Figure S17. Amino acid sequence of the BB0323$_{BG}$ monovalent recombinant protein. The sequence derived from the pUET1 vector is marked in black and the His-tag sequence within it is marked in green; the S-Tag domain in orange; fragment of the BB0323$_{BG}$ is marked in blue.

1-60 MHHHHHHSSG LVPRGSGMKE TAAAKFERQH MDSPDREDMK ILYSEIAKLR KNLNLNHLEI
61-120 DDILEKVAKE YAIKLGENKT LTHTLFGTTP MQRIHKYDKS FNLTREILAS GIELNRVVDA
121-180 WLNSPSHKEA LINTDTTKIG GYRLKTDNNI NIFVVLFGKR KDLGTDDDDK SPGFSSTMAI
181-202 SDPNSSSVDK LAAALEHHHH HH

Figure S18. Amino acid sequence of the BB0689$_{BA}$ monovalent recombinant protein. The sequence derived from the pUET1 vector is marked in black and the His-tag sequence within it is marked in green; the S-Tag domain in orange; fragment of the BB0689$_{BA}$ is marked in blue.

1-60 MHHHHHHSSG LVPRGSGMKE TAAAKFERQH MDSPDREDMK ILYSEIAELR KKLNLNHLEI
61-120 DDTLEKVAKE YAIKLGENRT ITHTLFGTTP MQRIHKYDQS FNLTREILAS GIELNRVVNA
121-180 WLNSPSHKEA LINTDTDKIG GYRLKTTDNI DIFVVLFGKR KDLGTDDDDK SPGFSSTMAI
181-202 SDPNSSSVDK LAAALEHHHH HH

Figure S19. Amino acid sequence of the BB0689$_{BB}$ monovalent recombinant protein. The sequence derived from the pUET1 vector is marked in black and the His-tag sequence within it is marked in green; the S-Tag domain in orange; fragment of the BB0689$_{BB}$ is marked in blue.

1-60 MHHHHHHSSG LVPRGSGMKE TAAAKFERQH MDSPDREDMK ILYSEIAELR KKLNLNHLEI
61-120 DDTLEKVAKE YAIKLGENKT LTHTLFGTSP MTRIHKYDKS FNLTREILAS GIELDRVVDA
121-180 WLKSPSHKEA LINKDTDKIG GYRLKTNNNI NIFVVLFGKR KDLGTDDDDK SPGFSSTMAI
181-202 SDPNSSSVDK LAAALEHHHH HH

Figure S20. Amino acid sequence of the BB0689$_{BG}$ monovalent recombinant protein. The sequence derived from the pUET1 vector is marked in black and the His-tag sequence within it is marked in green; the S-Tag domain in orange; fragment of the BB0689$_{BG}$ is marked in blue.

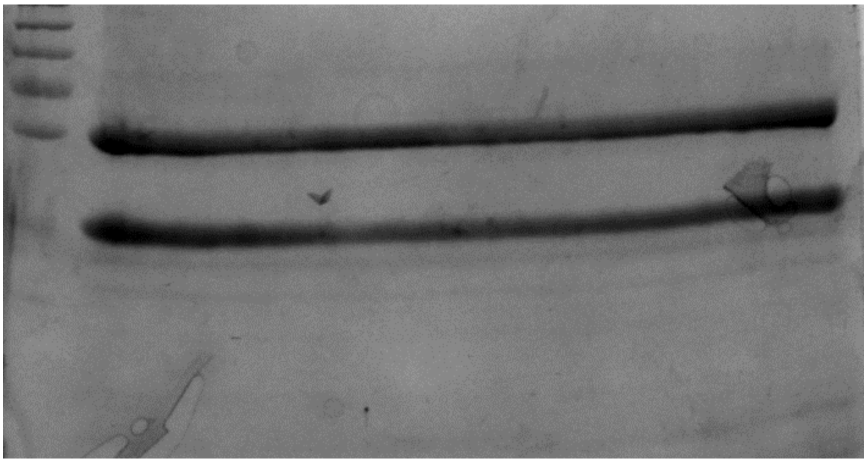

Figure S21. SDS-PAGE of *B. burgdorferi* s.l recombinant proteins in polyacrylamide gel prepared with the use of modified combs.

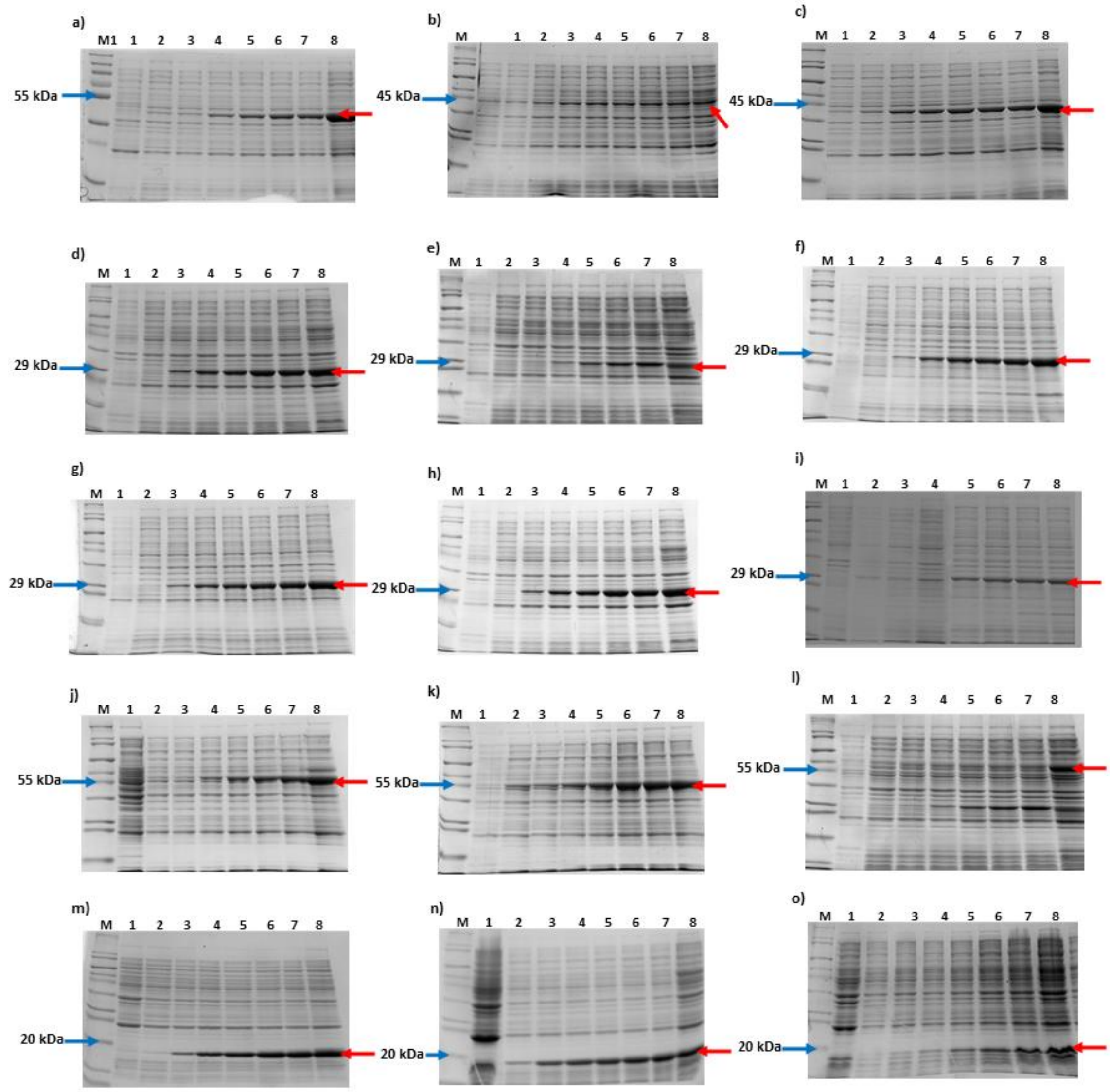

Figure S22. Production of recombinant proteins. SDS-PAGE of proteins contained in *E. coli* BL21(DE3)pLysS whole cell lysates; (LB, 37°C). The bands corresponding to the putative recombinant proteins are marked with red arrows: a) BB0108$_{BA}$; b) BB0108$_{BB}$; c) BB0108$_{BG}$; d) BB0126$_{BA}$; e) BB0126$_{BB}$; f) BB0126$_{BG}$; g) BB0298$_{BA}$; h) BB0298$_{BB}$; i) BB0298$_{BG}$; j) BB0323$_{BA}$; k) BB0323$_{BB}$; l) BB0323$_{BG}$; m) BB0689$_{BA}$; n) BB0689$_{BB}$; o) BB0689$_{BG}$.

Lanes:

M1 - PageRuler Prestained Protein Ladder

M - Protein marker, SigmaMarker™

1 - Negative control, *E. coli* BL21(DE3)pLysS + pUET1

2 - *E. coli* BL21(DE3)pLysS + appropriate recombinant plasmid, before induction

3 - *E. coli* BL21(DE3)pLysS + appropriate recombinant plasmid, 1 h after induction

4 - *E. coli* BL21(DE3)pLysS + appropriate recombinant plasmid, 2 h after induction

5 - *E. coli* BL21(DE3)pLysS + appropriate recombinant plasmid, 3 h after induction

6 - *E. coli* BL21(DE3)pLysS + appropriate recombinant plasmid, 4 h after induction

7 - *E. coli* BL21(DE3)pLysS + appropriate recombinant plasmid, 5 h after induction

8 - *E. coli* BL21(DE3)pLysS + appropriate recombinant plasmid, 18 h after induction

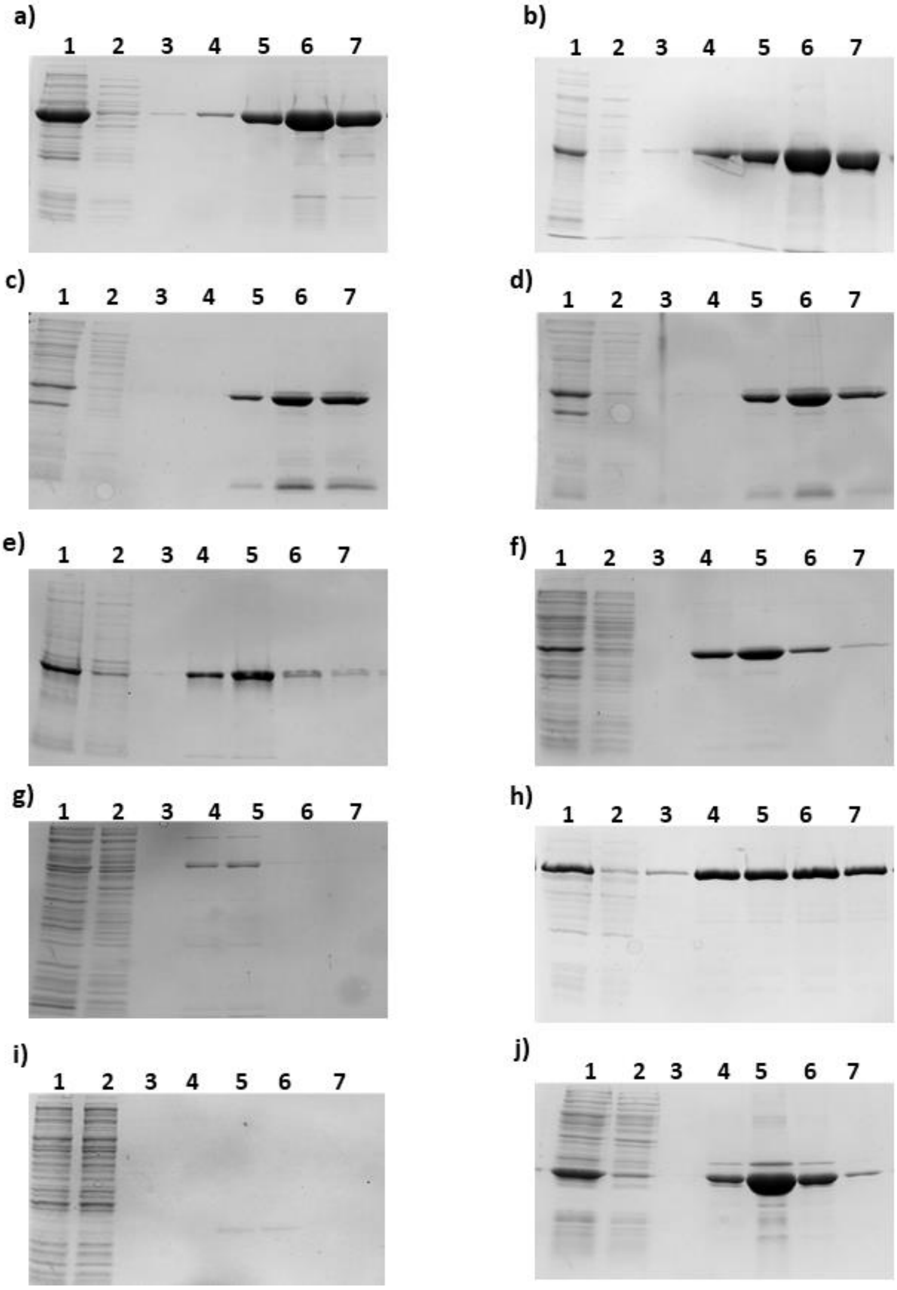

Figure S23. Results of purification of recombinant proteins: a) BB0108$_{BA}$ (buffers without urea); b) BB0108$_{BA}$ (buffers with urea); c) BB0126$_{BA}$ (buffers without urea); d) BB0126$_{BA}$ (buffers with urea); e) BB0298$_{BA}$ (buffers without urea); f) BB0298$_{BA}$ (buffers with urea); g) BB0323$_{BA}$ (buffers without urea); h) BB0323$_{BA}$ (buffers with urea); i) BB0689$_{BA}$ (buffers without urea); j) BB0689$_{BA}$ (buffers with urea).

Lanes:
1 - *E. coli* BL21(DE3)pLysS lysate before loading into the affinity column
2 - E. coli BL21(DE3)pLysS lysate after passing through the affinity column
3 - *First* elution fraction
4 - Second elution fraction
5 - Third elution fraction
6 - Fourth elution fraction
7 - Fifth elution fraction
